# Target labelling for the detection and profiling of microRNAs expressed in CNS tissue using microarrays

**DOI:** 10.1186/1472-6750-6-47

**Published:** 2006-12-12

**Authors:** Reuben Saba, Stephanie A Booth

**Affiliations:** 1 Division of Host Genetics & Prion Diseases, National Microbiology Laboratory, 1015 Arlington Street, Public Health Agency of Canada, Winnipeg, MB, R3E 3R2, Canada; 2 Department of Medical Microbiology and Infectious Diseases, Faculty of Medicine, University of Manitoba, Winnipeg, MB, R3B 1Y6, Canada

## Abstract

**Background:**

MicroRNAs (miRNA) are a novel class of small, non-coding, gene regulatory RNA molecules that have diverse roles in a variety of eukaryotic biological processes. High-throughput detection and differential expression analysis of these molecules, by microarray technology, may contribute to a greater understanding of the many biological events regulated by these molecules. In this investigation we compared two different methodologies for the preparation of labelled miRNAs from mouse CNS tissue for microarray analysis. Labelled miRNAs were prepared either by a procedure involving linear amplification of miRNAs (labelled-aRNA) or using a direct labelling strategy (labelled-cDNA) and analysed using a custom miRNA microarray platform. Our aim was to develop a rapid, sensitive methodology to profile miRNAs that could be adapted for use on limited amounts of tissue.

**Results:**

We demonstrate the detection of an equivalent set of miRNAs from mouse CNS tissues using both amplified and non-amplified labelled miRNAs. Validation of the expression of these miRNAs in the CNS by multiplex real-time PCR confirmed the reliability of our microarray platform. We found that although the amplification step increased the sensitivity of detection of miRNAs, we observed a concomitant decrease in specificity for closely related probes, as well as increased variation introduced by dye bias.

**Conclusion:**

The data presented in this investigation identifies several important sources of systematic bias that must be considered upon linear amplification of miRNA for microarray analysis in comparison to directly labelled miRNA.

## Background

MicroRNAs (miRNA) are an evolutionarily conserved, large new class of ~22 nucleotide (nt) long, gene regulatory RNA molecules that are involved in silencing mRNA transcripts through sequence-specific hybridization to 3' UTRs of mRNA molecules [1]. In plants, gene silencing is mediated primarily through RNA interference where the miRNAs are fully complementary to their mRNA targets. In contrast, animal miRNAs are only partially complementary to their targets, and silence gene expression by mechanisms that involve the co-localization of miRNAs and miRNA targets to cytoplasmic foci known as P-bodies as well as degradation of target mRNA [2-8]. Concurrently, a role for miRNAs in proliferative diseases has also been suggested, specifically during cancers, where a large number of miRNAs appear to be de-regulated in primary human tumours [9-13]. The current paradigm that miRNAs represent a new layer of gene regulation has generated much interest in this field. Thus, detection of miRNAs, their expression analysis, and identification of potential regulatory targets (cognate mRNA) are burgeoning areas of research.

The most commonly used technique to detect miRNAs is a Northern blot. A Northern blot can reliably profile the transcription of miRNAs and has often been used in the analysis of developmental and tissue-specific expression patterns [18-22]. However, this method is also limited because it cannot be used for the simultaneous monitoring of hundreds of miRNAs and requires substantial amounts of sample. As such, microarray technology provides a promising alternative to the Northern blot as numerous miRNAs can be analyzed at once with relatively minimal amount of initial RNA investment [23].

A number of recent reports have outlined ways in which microarray technology can be used to detect and profile the expression of miRNAs isolated from cells or tissues [15,16,24-35]. These reports can be classified into several categories based on variations in the methodologies employed to prepare labelled-targets for hybridization. Firstly, there are reports in which the mature ~22 nt long miRNAs have been directly labelled and used for hybridization [24,28,30,33-35]. Secondly, reports in which cDNA synthesized from the reverse transcription of adaptor-ligated miRNAs have served as the labelled miRNA targets for hybridization [15,26,29,31]. In this category, either modified bases capable of binding or already containing a label have served as nucleotides for cDNA synthesis during reverse transcription, or the adaptor-specific primers used for reverse transcription were the source of the label for the miRNA. Thirdly, there are reports that are similar to the second category except that the cDNA is PCR amplified prior to serving as the targets for hybridization [16,25,27,32]. In this category, the miRNAs were initially ligated to specific adaptors at both the 3' and 5' ends. Additionally, there are other novel methods that are presently being developed to measure miRNA expression [16,36].

From the survey of published reports it is apparent that miRNA microarray analysis is a growing field but it is also in its infancy and there is a need for detailed comparison of data obtained from different methodologies before a consensus is drawn on the ideal method(s) for labelled target preparation. Based on this premise and our ultimate goal to analyze very limited amounts of miRNA obtained from procedures such as laser capture microdissection, we evaluated two different methods for the preparation of labelled-targets; targets prepared from amplified and non-amplified miRNA. The amplification of limited amounts of RNA prior to microarray analysis is a common strategy for longer transcripts; however, less is known about the effects of amplification of miRNAs for microarray analysis.

The primary aim of this investigation was to optimize a simple and dependable labelling protocol for microarray analysis of miRNAs from enriched mixtures of low molecular weight (LMW) RNA without gel purification that would have potential for use when the starting quantity of RNA is low. Our objectives for this investigation were; to determine whether the sensitivity of detection of miRNAs can be increased by amplification as compared to a direct labelling methodology, and to determine whether an accurate representation of the original miRNA population is retained following amplification.

## Results

### Construction and specificity of the miRNA microarray

A total of 557 unique oligonucleotide probes to the majority of metazoan miRNAs in the miRNA registry (Release 5.0) [37] as well as to mature miRNA sequences from published literature at the time the project was initiated, were spotted onto glass slides in duplicate grids (See Additional File 1). Probes are in the same orientation as the miRNAs themselves. An initial evaluation showed that modifications of the probes to covalently link them to the slide surface was not necessary in order to allow high signal intensities with our choice of surface chemistry, epoxide coated slides from Corning (data not shown). We did however find that the addition of a 15 nucleotide poly(T) tract at the 5' end of the probes increased signal intensities, possibly by reducing steric hindrance due to the proximity of the short probe sequences to the slide surface.

**Figure 1:**
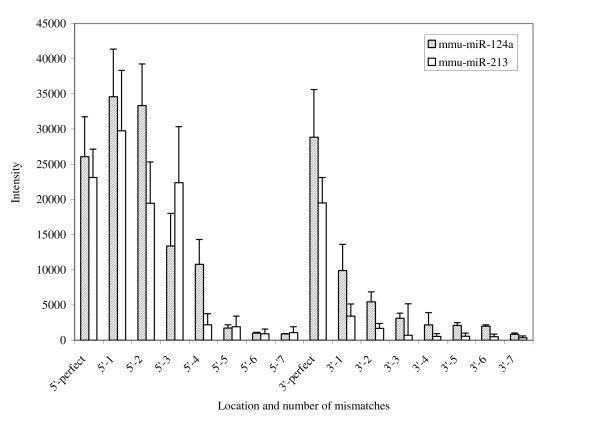
**Specificity of the complementary miRNA microarray probes**. Synthetic miRNA targets were hybridized to arrays containing probes that had increasing number of nucleotide mismatches at either the 3' or 5' ends.

We tested the specificity of the probes on the microarray by designing matched and mismatched probes and comparing the signal intensities after hybridization with labelled miRNAs. For this, an array was constructed on which oligonucleotide probes for two different miRNAs were spotted, additional probes were then designed around these sequences containing increasing number of nucleotide mismatches. All probes were spotted in quadruplicate on each slide. Along with probe specificity, the relationship between the 3' and 5' location of the mismatches was also assayed. At most, there were 7 nucleotide mismatches originating at either the 3' or 5' end of a probe; probe sequences are provided in Table 1. Complementary, synthetic miRNA targets were then hybridized to these arrays under standard conditions (overnight incubation at 50°C). The results are displayed in Figure 1. We found that mismatches at the 5' end of the probes for the miRNA sequences we looked at were tolerated to a significantly greater extent than those at the 3' end. In both cases, up to 2 mismatches at the 5' end did not result in any significant decrease in hybridization, with up to 4 mismatches giving hybridization signal above background. Signal was significantly reduced upon hybridization of both types of miRNAs to probes containing only one or two mismatches at the 3' end; signal was less than half that of perfectly matched probe and target. Signal was not above background when 3 or more mismatches were located at the 3' end of the probe. We also varied the hybridization temperature (42, 46, 55°C) to monitor the effect on array specificity, however, little change in specificity was apparent at the different temperatures assayed.

From this data we do not expect the microarray to consistently resolve miRNAs that have only one or two base differences, especially towards the 5' end. The majority of identified miRNAs, however, differ from each other by 5 or more mismatches and so in most of the cases we can confidently discriminate between different miRNAs [25]. Closely related miRNAs may require further analysis by other means to accurately measure their levels of expression [38,39].

### Development of a linear amplification labeling method for preparation of antisense miRNA targets

**Figure 2:**
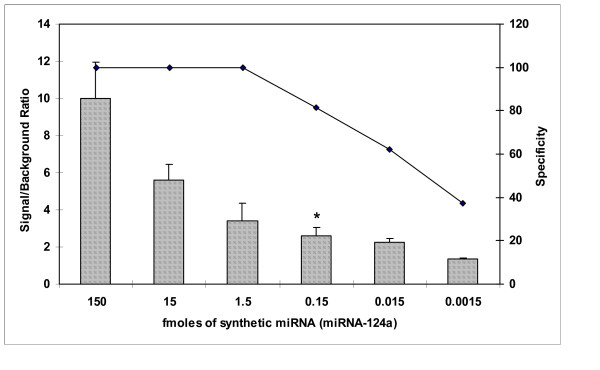
**Detection range of miRNAs by the linear-amplification strategy**. A synthetic miRNA (miR-124a) was titrated, linear amplified, and hybridized to the arrays. Signal to background ratio and specificity of binding of the miRNA is shown. The amount of miRNA that can be reliably amplified and detected by the microarray is denoted with an asterisk.

We developed a method to prepare and label miRNA targets using a linear amplification procedure in which an antisense-labelled target is produced. RNA is polyadenylated and used as a template for cDNA production. After second strand synthesis a T7 linear amplification protocol was used to produce antisense RNA that was conjugated with fluorescent dyes prior to hybridization to microarrays (See Additional File 2). Using 100 ng of LMW RNA as starting material a strong fluorescent signal was detected, with some spots close to saturation, and we went on to use this labelling methodology to test a range of concentrations of miRNA starting material to determine the sensitivity threshold, and the dynamic range, for detection of our microarray, after target amplification. This was done by titration of a commercially synthesized miRNA and performing linear amplifications on the titrations to generate amplified labelled-targets (labelled-aRNA). The labelled-targets were then hybridized to arrays that had complimentary probes printed in quadruplicate. Unrelated miRNAs and probes with between 1 and 7 nucleotide mismatches were printed as specificity and hybridization controls. The signal/background ratios for hybridizations using between 150 fmoles to 1.5 amoles of miRNA target was calculated and shown in Figure 2. Background was determined as an average of intensities from spots containing an unrelated control miRNA probes. We found that 150 amoles of miRNA can be reliably amplified and detected by our microarray. We detected signal on the probes even after amplification of only 1.5 amoles of miRNA, but we found that as we lowered the quantity of miRNA, the signal from mismatched probes increased relative to the perfectly matched probes. We calculated this reduction in specificity based on the ability to discriminate between hybridization to perfectly matched probes, and those containing 5 mismatches, and plotted these values on Figure 2. We concluded that errors are introduced into the miRNA targets during the amplification procedure resulting in loss of hybridization specificity. The microarray had a broad range of detection of approximately three orders of magnitude.

### Confirmation of the validity of miRNA expression profiles following amplification

We went on to determine whether amplification affects the proportional representation of miRNA species identified from a complex mixture, in comparison to another method that does not include an amplification step.

Expression profiles for miRNAs isolated from brain tissue and amplified using the T7 linear amplification method as previously described were compared with profiles generated using antisense, tagged-cDNA hybridized to the array and signal amplified by dendrimer technology (See Additional File 2). The starting population of LMW RNA for all experimental procedures was prepared from total mouse brain as a single batch to ensure reproducibility. Each labelling reaction used between 100 and 250 ng of this LMW RNA as outlined in the methods section. The only variable factor was the labelled miRNA preparation technique employed. Total RNA consists of ~0.1% miRNA and the miRNA isolation technique we used results in a 10 fold enrichment of small RNAs (less than 200 nt long) and so between 1 ng and 2.5 ng of miRNA was used in each labelling reaction.

Significant signal was detectable on negative control spots, as well as to miRNA probe sequences from species other than mice, and not present in the mouse genome. We adjusted the intensity values for the spots to take this into account; an average signal intensity value was calculated for each slide from these control spots, and this was then subtracted from all spots on the array to determine net signal intensities. In contrast, hybridizations of target LMW RNA prepared without an amplification step (labelled-cDNA) had lower signal intensities, but little detectable non-specific hybridization to control spots; in this case the background correction step was omitted. The detection limit for miRNA labelled by the direct labelling technique was approximately 1.5 fmoles and the detection range was approximately 2.5 orders of magnitude as determined by serial dilution, labelling and hybridization of the synthetic miRNA (data not shown).

**Figure 3:**
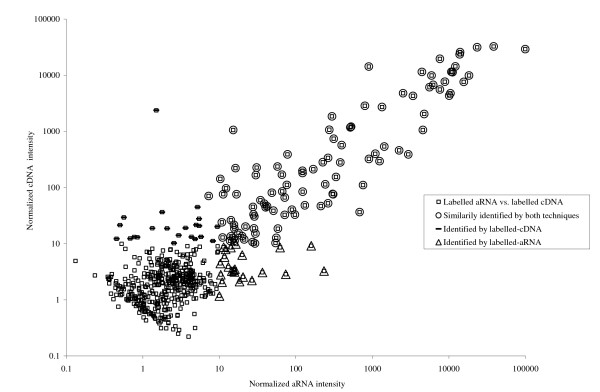
**A representative correlation scatter plot of the normalized signal intensities for the labelled-aRNA versus the directly labelled-cDNA**. Open circles represent miRNAs identified by both techniques when all spots having normalized intensities less than 10 were scored as absent.

**Figure 4:**
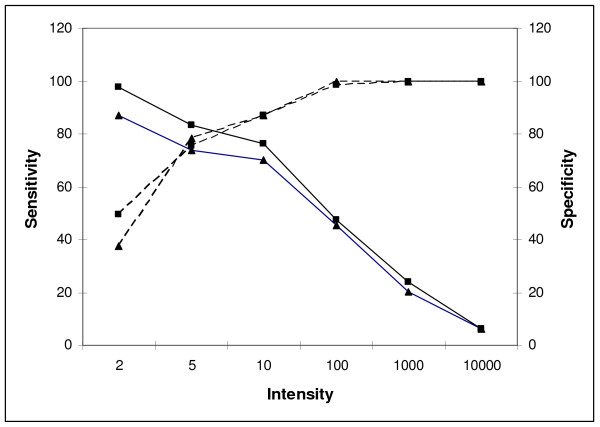
**Sensitivity and specificity plot of detection of amplified RNA targets and labelled cDNA targets versus the detection threshold**. Sensitivity was determined by – number of true positives/(number of true positives + number of false negatives) for a number of detection thresholds using the Taqman^® ^results for comparison. Specificity was similarly calculated as – number of true negatives/(number of true negatives + number of false positives). ▲ denotes labelled-aRNA; ■ denotes labelled-cDNA; ---- denotes specificity; — denotes sensitivity.

Individual miRNAs were then scored as being present or absent based on the presence of a reproducible signal in three replica hybridizations, for each of the labelling techniques evaluated. A representative scatter plot illustrating this data is shown in Figure 3. We determined that a core group of 100 (~18%) spots on the array were scored as present when either of the target labelling techniques was used. In addition, 22 miRNAs were reproducibly detected on our array only when cDNA labelled targets were used, with a further 23 different miRNAs only with the aRNA labelled targets. The correlation coefficients (R^2^) for aRNA labelled targets versus cDNA labelled targets through repeated measurements were low, 0.58 ± 0.15 (n = 5). However, we concluded that in general, the two target labelling techniques evaluated show a high degree of reproducibility in regards to the miRNAs scored as present by both methods. A plot of sensitivity and specificity for each labelling method is provided in Figure 4. Although we found that the sensitivity of detection for amplified miRNAs was at least one log higher than for direct labelled miRNAs using labelling of synthetic miRNAs i.e. 150 amols for amplified miRNAs versus 1.5 fmols for directly labelled miRNAs, this was not the case in this experiment using total LMW RNA as starting material. Sensitivity and specificity measurements were in this case very similar when either labelling method was used. We concluded that the decrease in specificity observed when starting with less abundant miRNAs, 150 amoles or lower, as recorded in Figure 2, contributed to the high non-specific hybridization signals that we observed using amplified RNA. This high background signal effectively masked any increase in sensitivity that might have resulted from amplification.

**Figure 5:**
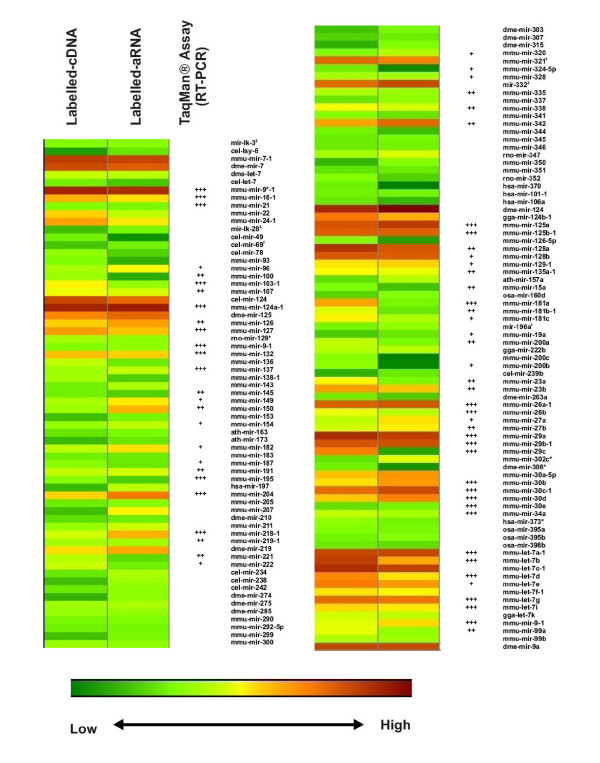
**Heat map of signal intensities from miRNAs that were similarly identified by both types of labelling techniques**.(†) Represents miRNAs that are no longer listed in the miRNA registry however present on the custom microarray. (‡) Represents miRNA-like elements from references 41 and 47. We scored TaqMan detected miRNA probes as present using the following criteria. Ath-miR-159a, cel-lin-4, and cel-miR-2 were used as the negative controls (Ct ~30–35). The Ct values used to determine +, ++, +++ expression patterns are as follows: +++ – 15–18; ++ – 18 – 21; + – 21–27;

The expression profile of these 145 miRNAs is illustrated in Figure 5 by a heat map of signal intensities of miRNAs determined to be present in mouse brain by hybridization of each of the labelled targets with our miRNA microarrays. At least 95 of the 145 miRNA sequences detected by either labelling methodology, using our array, were predicted to be expressed in mouse tissues [37]. This number represents approximately two thirds of the miRNAs estimated to be expressed in mouse tissues from information in the miRNA registry (Release 5.0). Significant tissue tropism has been associated with many miRNAs and we would therefore not expect to detect all mouse miRNAs in brain tissue. A comparison with previous literature on tissue tropism of miRNA expression shows a high degree of correlation for brain expressed miRNA profiles obtained using our miRNA microarrays to those obtained by Northern blotting. In particular, comparison with the comprehensive study by Sempere *et al*. showed 79% similarity in the miRNAs assayed and called as present in mouse brain tissue [22]. The majority of the remaining 21% were of low intensity and may not have been detectable by Northern Blot.

### Validation of microarray data by qRT-PCR

A third method, qRT-PCR was used for independent validation of miRNA microarray data for the relative expression of miRNAs in mouse brain [39]. This method has been determined to be quantitative and sensitive; it requires as little as 50–100 ng of total cellular RNA as starting material and is specific enough to allow for discrimination between miRNAs differing by a single nucleotide. We determined the dynamic range of the qRT-PCR assay was from 1.5 pmoles to 1.5 amoles for miRNAs using a dilution series of the synthetic miRNA previously described.

**Figure 6:**
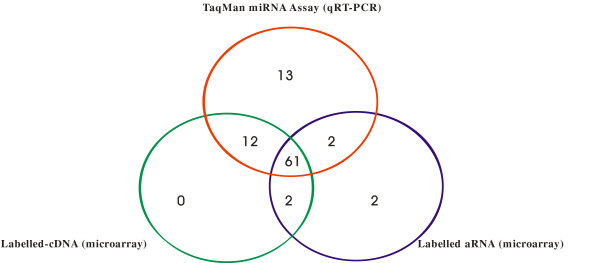
Venn diagram to illustrate miRNAs detected by TaqMan^® ^MiRNA Assay quantification and miRNA microarray analysis.

In total, 157 of the miRNAs probes on the microarray were validated using this assay. Positive (miR-16, let-7a) and negative (ath-miR-159a, cel-miR-2, cel-lin-4) controls used in the qRT-PCR assay were used to assign Ct cut-off values to assign present or absent calls for each miRNA. The negative control miRNAs represent miRNAs not expressed in mouse tissue and we determined the Ct values for these probes to be on average >30 for an input amount of 20 ng LMW RNA. MiRNAs scored as either present (Ct<27) or absent were then compared with microarray data; 75 of the miRNAs chosen for validation were also scored as being present by microarray analysis and many of these are indicated in Figure 5 for comparison. A direct comparison between all the probes validated by qRT-PCR and by microarray for both types of target labelling attempted are concisely summarised in Figure 6.

The qRT-PCR assay proved to be the most sensitive method for the detection of miRNAs with 88 being positively detected in comparison to the microarray; 75 miRNAs detected with the cDNA labelled targets and 67 for the amplified targets. Amplification of miRNAs, in this case, did not result in increased sensitivity for low abundance miRNAs; probably due to the high background when using the amplified target which masked low intensity signals. The high background is likely due to non-specific hybridization of incomplete reverse transcription, or template-independent, products produced during the amplification steps that bind with less specificity than their full-length counterparts [31,40].

With the exception of miRNAs not scored as present by microarray analysis, the results obtained from each of the methods evaluated were remarkably similar. Only 2 miRNAs were scored as present by microarray analysis using targets labelled by either the amplification or direct labelling techniques but not by the qRT-PCR assay. In turn, these 2 miRNAs had intensities that were very close to the cut-off values for the scoring of miRNAs as being present based on array signals; in fact 61 miRNAs were similarly scored as present by each method showing a high degree of agreement between the three methods evaluated.

We concluded from these results that although the sensitivity of microarray detection was lower than qRT-PCR, the accuracy of miRNA detection, however, was comparable within the detection range of the arrays. In addition, we found that the amplification step did not significantly alter the proportional representation of miRNAs *in vivo*, especially for those miRNAs present in high abundance. It is readily apparent from the expression map that the most abundant miRNAs, including brain specific or enriched miRNAs, miR-124a-1, -9-1, -9*-1, -127, -136, -138-1, -149, -154, -218-1, -219-1, -222, -125a, -125b-1, -128a, -26a-1, -29a, -29b-1, -30c-1, and -34a were equally identified by both types of microarray labelling techniques. Amongst the abundant miRNAs identified, detection of miR-124a-1 was especially important as it is often cloned at very high frequencies in the brain [18,25,41]. Furthermore, miR-124a-1 has been known to down-regulate approximately 174 genes in transfected HeLa cells and in the process alters the entire transcriptome to a neuronal-like mRNA profile [42].

### Dye bias associated with amplification and direct labelling methods

**Figure 7:**
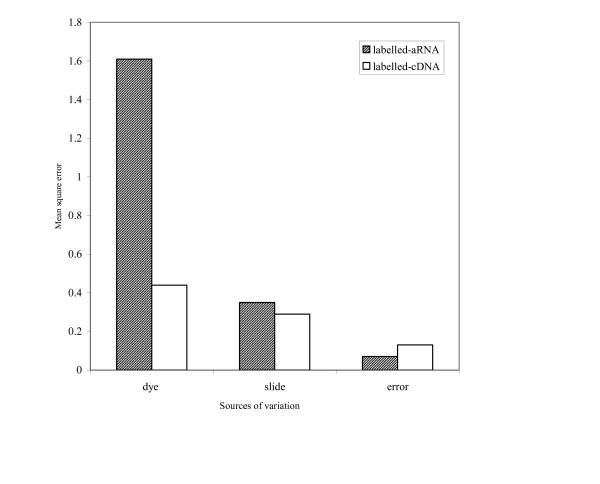
Estimates of variation introduced by dyes and technical replication by an ANOVA analysis.

Dye bias is a contributing factor to technical variation seen between replicas in microarray experiments; this is the appearance of consistently brighter signals for individual targets in either the green or red channel when there are no differences in expression between the two. We were interested to examine whether the amplification procedure in particular contributed to this effect in the analysis of miRNAs by our microarrays. We performed hybridizations to 8 microarrays using a single batch of LMW RNA prepared from mouse brain. The LMW RNA was split into aliquots, and either amplified for labelled-aRNA or direct-labelled for cDNA. For each technique evaluated, half of the RNA was then labelled with either the red or green dye and aliquots hybridized to arrays (n = 6). An ANOVA analysis to estimate the variation introduced by the dye and the slide (technical replication) was performed. The results are illustrated in Figure 7. It was apparent from the results that the slide-to-slide variation is similar for each method, but that the variation introduced by the dye was significantly larger for the targets prepared as labelled-aRNA than for the direct-labelled targets.

The dye bias in favour of amplified targets may be due to the variability of incorporation kinetics and quenching susceptibilities of modified dNTPs in the amplification reactions, thus altering fluorescent signals [43]. Normalization by lowess smoothing did not effectively remove dye bias from these self-self hybridizations. In contrast, the significantly lower dye bias for labelled-cDNA may be due to the dendrimer geometry which permits equal spacing between adjacent dye molecules thus preventing energy transfer and other types of quenching interactions which often reduce fluorescent signal [44]. Furthermore, each cDNA transcript binds a single dendrimer and each dendrimer has a predetermined number fluorescent molecules. Cy3-labelled dendrimers have on average of 250 fluorescent molecules per dendrimer whereas the Cy5-labelled dendrimers have 243 flours per dendrimer, with a standard error of ± 4 molecules. Recent improvements in denrimer technology have resulted in nearly 900 fluorescent molecules per dendrimer. It was concluded that labelling transcripts for detection via dendrimers is likely a more reproducible methodology for quantitative assessment of miRNAs.

### Validation of microarray derived expression ratios using qRT-PCR

To determine the utility of the miRNA microarray to quantitatively measure expression ratios we performed experiments to identify differentially expressed miRNAs in a number of brain-derived cell lines. The cell lines used were three lines with microglial characteristics (EOC 13.31, EOC 20, C8-B4), and four lines derived from neuroblastomas (NB41A3, SK-N-FI, IMR-32, Neuro-2a). Each cell line was cultured and passaged in the laboratory, and LMW RNA was isolated from a number of different passages so that four biological replicates were produced from each line. LMW RNA was isolated from these cells and used to produce labelled cDNA; these samples were then hybridized to the miRNA arrays. Arrays were scanned and the intensity values obtained were used for further analysis. Consistently differentially expressed miRNAs between cell lines of microglial and neuroblastoma origin were selected by the 'significance analysis of microarrays' (SAM) [45] two-class analysis. We identified 68 miRNAs to be significantly differentially expressed between these two groups of cell lines with an average fold change over 2. We picked two miRNAs whose expression differed most significantly between the two groups of cell lines to examine more closely. The expression levels in each of the cell lines were compared and validated using qRT-PCR (TaqMan^® ^MicroRNA Reverse Transcription Assay from Applied Biosystems). In order to directly compare expression levels from each method we chose the Neuro-2A line as a 'standard' for comparison, and calculated the fold changes between this line and all the others using either, microarray signal intensities, or qRT-PCR assay. The results are summarised in Table 2. We show there to be a high degree of concordance between the expression levels calculated for each of the miRNAs by either method. MiR-222 is expressed at significantly higher levels in cell lines of microglial origin over those of neuroblastoma origin, and vice versa for miR-137. Smaller changes in expression were also picked up between individual cell lines using both methods. As has been found previously in experiments to measure differential expression in mRNAs, the fold changes calculated by qRT-PCR are often greater than those calculated by microarray. The validation of expression trends derived from analysis with our miRNA microarrays using quantitative RT-PCR provides a high degree of confidence in our use of these arrays to measure expression trends.

## Discussion

The primary aim of this investigation was to examine target labelling methodologies that could be used for our custom miRNA microarray that did not require labour intensive steps such as gel purification, were sensitive and specific, useable at high-throughput and more affordable than multiplex RT-PCR assay. In particular, we were interested to determine whether the amplification methodology routinely in use for mRNA profiling could be directly applied to the study of miRNAs. It was clear from our evaluation that high-throughput microarray analysis of miRNA expression could be performed accurately using amplified targets.

Investigation of the sensitivity of detection, using a synthetic miRNA and an array of matched and mismatched probes, we found there to be an advantage in terms of sensitivity of detection for low-abundance miRNAs after amplification, in comparison to a direct labelling technology. However, when specificity was examined on the same platform, we found that the ability to discriminate between closely related sequences was reduced after amplification.

Using total brain LMW RNA as starting material for both the direct labelling and amplification protocols we measured very similar sensitivity and specificity for both methods; a TaqMan^® ^quantitative RT-PCR method was used as the 'gold standard' for validation. We concluded that the reduction in specificity after amplification resulted in high non-specific signal intensities across the array, which masked detection of low abundance miRNAs. It is possible that by using an amplification technique, in which only full length targets are selectively labelled, to reduce non-specific hybridization, the sensitivity of detection for methods including amplification could be increased; potentially this increase in sensitivity could bring the range of detection for microarrays to a value similar to the 10 amoles detection limit that was observed for the qRT-PCR assay, and a requirement for μg rather than mg quantities of starting material. In this case, PCR amplification of reverse transcription products prior to linear amplification to select for full-length transcripts may be the solution to these problems [27]. Initially, this would require the ligation of specific adapters to both the 3' and 5' ends of the mature miRNAs and the use of adapter specific primers for the PCR reactions [27].

It is worth noting at this point, a new addition to microarray based analysis of miRNAs; the RNA-primed array-based Klenow enzyme (RAKE) assays [46]. In this technique, antisense DNA oligonucleotide sequences are used to probe for miRNAs and upon binding miRNAs serve as primers for Klenow enzyme based extension. During the generation of the double stranded fragment, tagged nucleotides are incorporated allowing for easy detection. The advantage of this method is that it does not require the manipulation of the initial RNA investment and also does not require the generation of a cDNA library or amplification. Most laboratories, however, have custom microarray platforms with optimized target labelling protocols and hopefully our results will alert those researchers in the field to possible areas of misinterpretation.

Lastly, we anticipate and encourage discussion about all aspects of microarray analysis of miRNAs, not only in the area of labelling but also in other areas of the technology where there is possibility for the introduction of systematic bias. High-throughput techniques in the analysis of miRNAs, such as microarrays, will be helpful to further understand the role(s) of miRNAs in normal and diseased cells and tissues.

## Conclusion

For high-throughput analysis of limited amounts of miRNAs by microarrays we show that linear amplification of LMW RNA targets does not significantly increase the sensitivity of low abundance miRNAs, in comparison to direct-labelled targets. Additionally, the amplified targets also showed decreased probe specificity and increased variation introduced by dye bias.

## Methods

### MiRNA microarray construction

A microarray containing oligonucleotide probes complimentary to 557 miRNAs was constructed. These probes consisted of all the non-redundant miRNA sequences from the miRNA registry (Release 5.0) [37]. The arrays were spotted in-house on Corning^® ^epoxide coated slides; Probes were spotted in duplicate and additionally two full arrays were printed per slide. The probes were in sense orientation relative to the mature miRNAs and thus complimentary to either the cDNA or aRNA that would be derived from the mature miRNAs. The probes also had a 15 nucleotide long poly(T) tract at the 5'end for steric distancing from the array surface. Each probe was printed in duplicate and the signal intensities for averaged.

### Low molecular weight RNA enrichment

Low molecular weight (LMW) RNA enrichment from whole mouse brain was performed using *mir*Vana™ miRNA Isolation Kit (Ambion), according to the manufacturer's protocol. LMW RNA was DNase-treated for microarray target preparation using TURBO DNA-*free*™ (Ambion). Chemically synthesized miRNAs were purchased from Dharmacon.

### Preparation of labelled-cDNA targets

Array 900 miRNA RT Kit (Genisphere) was used to prepare labelled cDNA targets for microarray hybridization according to the manufacturer's protocol. Briefly, 250 ng of LMW enriched RNA was used as a template in a 100 μl Poly(A) Tailing and RT reaction containing 1× Reaction Mix, 2.5 mM MnCl_2_, 1 mM ATP, and 4 μL poly A polymerase (*E*-PAP Enzyme). After incubation at 37°C for 15 minutes the reaction was placed on ice and 2 μL of Cy3 or Cy5 reverse transcriptase primer was added. The reaction mix was incubated at 65°C for 10 minutes prior to addition of 23 μL of a second master mix containing per reaction: 10 μL of 5× First Strand Buffer, 5 μL of 0.1 M DTT, 2.5 μL of dNTP Mix, 1 μL of Superase-in RNase Inhibitor, 2 μL of SuperScript II Reverse Transcriptase (200 U) (Invitrogen), and 2.5 μL of nuclease-free water. Subsequently, the reaction was incubated at 42°C for one hour. Finally, 8.75 μL of 0.5 M NaOH/50 mM EDTA and 65°C for 15 minutes was used to inactivate Superscript II. Samples were concentrated to a volume of approximately 10–15 μL, using Microcon YM-10 Centrifugal Filter Devices (Fisher Scientific) according to the manufacturer's protocol.

### Preparation of labelled-aRNA targets

LMW enriched RNA was polyadenylated using the Poly(A) Tailing Kit (Ambion); 250 ng of LMW enriched RNA was used as a template for a 25 μl Poly(A) Tailing reaction containing 1× Reaction Mix, 2.5 mM MnCl_2_, 1 mM ATP, and 1 μL poly(A) polymerase (PAP Enzyme). The reaction was placed on ice. For differential labelling of targets, approximately 100 ng of polyadenylated LMW RNA was mixed with 1 μL of T7 Oligo(dT) Primer, and nuclease-free water added to 12 μL. After incubation at 70°C for 10 minutes 8 μL of a reverse transcription mixture was added, 2 μL of 10 × First Strand Buffer, 1 μL Ribonuclease Inhibitor, 4 μL dNTP Mix, and 1 μL of Reverse Transcriptase, and incubation continued at 42°C for 2 hours. Following this 80 μL of second strand synthesis buffer was added, which included 1× Second Strand Buffer, 4 μL of dNTP Mix, 2 μL of DNA polymerase, and 1 μL of RNase H, and the sample incubated at 16°C for 2 hours. Newly synthesized cDNA was purified using cDNA Filter Cartridges and used for an *in vitro *transcription reaction to generate chemically modified aRNA. *In vitro *transcription was performed in a 40 μL reaction including the double stranded cDNA from the second strand synthesis, 3.75 mM aaUTP, 7.5 mM ATP, CTP, GTP Mix, 3.75 mM UTP, 1× T7 Reaction Buffer, and 4 μL of T7 Enzyme Mix. The mix was incubated for 14 hours at 37°C and then treated with DNase (2 μL per reaction) for 30 minutes at 37°C to remove template cDNA. The aRNA was purified using aRNA Filter Cartridges and the aRNA:dye coupling reaction was performed according to the manufacturer's protocol. Briefly, 5–20 μg of amino allyl aRNA was dried down to a thin film in a microfuge tube and 9 μL of coupling buffer, and 11 μL of either of the dyes Alexa Fluor Succinimidyl Ester 555 or 647 constituted in DMSO (one dye vial re-suspended in 88 μL of DMSO) (Invitrogen), were added. After incubating at room temperature for 30 minutes in the dark, 4.5 μL of 4 M hydroxylamine was added to quench the amine-reactive groups of the unreacted dye molecules. After incubation for 15 minutes at room temperature the labelled amino allyl aRNA was purified using aRNA filter cartridges.

### Target hybridization to microarray

Tagged-cDNA hybridization followed the protocol outlined in the 900 miRNA RT Kit. A hybridization mixture consisting of the differentially tagged cDNA (10 μL of Cy3-labelled and 10 μL of Cy5-labelled targets) and 2 × SDS-based Hybridization Buffer pre-heated to 70°C (20 μL) was mixed and incubated at 75–80°C for 10 minutes, cooled to 50°C until loading and added to the microarray; specifically a 22 × 40 mm cover slip (mSeries Lifterslip™) (Erie Scientific) was centered over the grids and the preheated hybridization mixture was loaded under the cover slip. Microarrays were incubated overnight (16–20 hours) at 50°C in a dark humidified chamber (Genetix). Following hybridisation the cover-slips were removed and the arrays were washed in 2 × SSC, 0.2% SDS wash buffer preheated to 42°C for 15 minutes, 2 × SSC wash buffer at room temperature for 10–15 minutes, and 0.2 × SSC wash buffer at room temperature for 10–15 minutes. Arrays were dried by centrifugation at 1000 rpm for 2–3 minutes and the 3DNA system containing the fluorescent cyanine molecules were hybridized to the arrays; in this case the hybridization mixture contained Cy3 3DNA Capture Reagent (2.5 μL), Cy5 3DNA Capture Reagent (2.5 μL), Nuclease Free Water (15 μL), and 2 × SDS-based Hybridization Buffer. The mix was heated to 70°C for 10 minutes, cooled to 62–64°C and hybridized to the arrays for 4 hours at 62–64°C in a dark humidified chamber. Finally, the arrays were washed as previously described.

Differentially labelled aRNA targets were hybridized to the arrays according to the protocol outlined in the Amino Allyl MessageAmp™ Kit. Since the purified labelled aRNA was eluted in a final volume of 100 μL, it was necessary to firstly concentrate the samples to approximately 1–5 μL by vacuum drying in the dark. aRNA target was resuspended in Pronto!™ Short Oligo Hybridization Solution (Corning) to a final volume of 20 μL and differentially labelled aRNA targets combined and loaded onto arrays. Hybridized arrays were incubated for 16 hours at 50°C in a dark humidified chamber. Upon incubation, the slides were washed in 2 × SSC, 0.1% SDS pre-warmed to 42°C for 5 minutes, twice in 1 × SSC wash buffer at room temperature for 2 minutes, and two successive washes in 0.1 × SSC at room temperature for one minute each time. The arrays were dried by centrifugation at 1000 rpm for 2–3 minutes.

### Microarray spot quantification and analysis

Microarrays were scanned using Agilent G2565AA and Agilent G2565BA Microarray Scanner System (Agilent). Feature extraction was performed using Array-Pro™ analyzer version 4.5 (Media Cybernetics). The intensities of duplicate spots on each array were averaged. Partek^® ^software, version 6.2 Copyright^© ^2006 (Partek) was used to perform ANOVAs.

### qRT-PCR (TaqMam^® ^MiRNA Assays)

Reverse transcriptase reactions were performed using the TaqMan^® ^MicroRNA Reverse Transcription Kit (Applied Biosystems). Each reaction contained 20 ng LMW enriched RNA, 1 mM dNTPs (with dTTP), 1 μL of 3.3 U MultiScribe™ Reverse Transcriptase, 1× Reverse Transcription Buffer, 3.75 U RNase Inhibitor, and 3 μL of RT primer. The reaction was carried out at 16°C for 30 minutes, 42°C for 30 minutes, and 85°C for 5 minutes. Semi-quantitative PCR reactions were performed according to the methodology outlined in the TaqMan^® ^MiRNA Assay Kit (Applied Biosystems). Briefly, each reaction contained TaqMan 1× Universal PCR Master Mix (No AmpErase UNG), 1× TaqMan^® ^MicroRNA Assay Mix, and 1.33 μL of the RT product in a total volume of 20 μL. Each reaction was incubated in an Applied Biosystems 7500 Real Time PCR System in a 96-well plate at 95°C for 10 minutes, followed by 40 cycles of 95°C for 15 sec and 60°C for 1 minute. The threshold cycle (Ct) method was used to determine the relative quantities of each miRNA, and was defined as the fractional cycle number at which the fluorescence passes the fixed threshold.

To determine the fold changes in expression between miRNAs in a number of cell lines we used the following methodology. The expression of each miRNA relative to let-7a was determined using the ΔΔCt method. Average fold differences for each miRNA were calculated by comparing the relative expression (ΔΔCt values) in each of the cell lines tested in comparison to a standard (ΔΔCt values were compared for each cell line EOC 13.31, EOC 20, C8-B4, NB41A3, SK-N-FI, IMR-32 with the ΔΔCt value for Neuro-2a). All experiments were performed in triplicate.

## Authors' contributions

RS performed the microarray experiments. SAB conceived and supervised the study. Both SAB and RS participated in the analysis of the data and drafting of the manuscript.

## Supplementary Material

Additional File 1 Oligonucleotide sequences of miRNA microarray probes. Click here for file

Additional File 2 Schematic comparison between the direct labelling strategy (labelled-cDNA) and labelling upon linear amplification (labelled-aRNA). Click here for file

## Figures and Tables

**Table 1:** Sequences of the miRNA probes spotted on the specificity microarrays

miRNA annotation^a^	Probe sequence 5'-3'	Number of mismatches
mmu-miR-124a [perfect]	ttttttttttttttttaaggcacgcggtgaatgcc	0
mmu-miR-124a [5'-1]	ttttttttttttttt**c**aaggcacgcggtgaatgcc	1
mmu-miR-124a [5'-2]	ttttttttttttttt**c**a**c**ggcacgcggtgaatgcc	2
mmu-miR-124a [5'-3]	ttttttttttttttt**c**a**c**g**a**cacgcggtgaatgcc	3
mmu-miR-124a [5'-4]	ttttttttttttttt**c**a**c**g**a**c**c**cgcggtgaatgcc	4
mmu-miR-124a [5'-5]	ttttttttttttttt**c**a**c**g**a**c**c**c**t**cggtgaatgcc	5
mmu-miR-124a [5'-6]	ttttttttttttttt**c**a**c**g**a**c**c**c**t**cg**t**tgaatgcc	6
mmu-miR-124a [5'-7]	ttttttttttttttt**c**a**c**g**a**c**c**c**t**cg**t**t**t**aatgcc	7
mmu-miR-124a [3'-1]	ttttttttttttttttaaggcacgcggtgaatgc**g**	1
mmu-miR-124a [3'-2]	ttttttttttttttttaaggcacgcggtgaat**c**c**g**	2
mmu-miR-124a [3'-3]	ttttttttttttttttaaggcacgcggtg**g**at**c**c**g**	3
mmu-miR-124a [3'-4]	ttttttttttttttttaaggcacgcgg**g**g**g**at**c**c**g**	4
mmu-miR-124a [3'-5]	ttttttttttttttttaaggcacgc**c**g**g**g**g**at**c**c**g**	5
mmu-miR-124a [3'-6]	ttttttttttttttttaaggcac**c**c**c**g**g**g**g**at**c**c**g**	6
mmu-miR-124a [3'-7]	ttttttttttttttttaag**c**cac**c**c**c**g**g**g**g**at**c**c**g**	7
mmu-miR-213 [perfect]	tttttttttttttttaccatcgaccgttgattgtacc	0
mmu-miR-213 [5'-1]	ttttttttttttttt**t**ccatcgaccgttgattgtacc	1
mmu-miR-213 [5'-2]	ttttttttttttttt**t**c**g**atcgaccgttgattgtacc	2
mmu-miR-213 [5'-3]	ttttttttttttttt**t**c**g**a**g**cgaccgttgattgtacc	3
mmu-miR-213 [5'-4]	ttttttttttttttt**t**c**g**a**g**cga**g**cgttgattgtacc	4
mmu-miR-213 [5'-5]	ttttttttttttttt**t**c**g**a**g**cga**g**cgttg**t**ttgtacc	5
mmu-miR-213 [5'-6]	ttttttttttttttt**t**c**g**a**g**cga**g**cg**g**tg**t**ttgtacc	6
mmu-miR-213 [5'-7]	ttttttttttttttt**t**c**g**a**g**cga**g**cg**gg**g**t**ttgtacc	7
mmu-miR-213 [3'-1]	tttttttttttttttaccatcgaccgttgattgtac**g**	1
mmu-miR-213 [3'-2]	tttttttttttttttaccatcgaccgttgattgt**t**c**g**	2
mmu-miR-213 [3'-3]	tttttttttttttttaccatcgaccgttg**g**ttgt**t**c**g**	3
mmu-miR-213 [3'-4]	tttttttttttttttaccatcgaccg**c**tg**g**ttgt**t**c**g**	4
mmu-miR-213 [3'-5]	tttttttttttttttaccatcga**t**cg**c**tg**g**ttgt**t**c**g**	5
mmu-miR-213 [3'-6]	tttttttttttttttaccatc**t**a**t**cg**c**tg**g**ttgt**t**c**g**	6
mmu-miR-213 [3'-7]	tttttttttttttttaccat**gt**a**t**cg**c**tg**g**ttgt**t**c**g**	7

a. The numbers in the square parenthesis represent the location and number of mismatches on the probes.

The nucleotide bases in bold represent the substitutions that were made to the original miRNA sequence.

**Table 2:** Comparison of miRNA fold changes in a number of brain derived cell lines by Microarray and RT-PCR

**Cell line**	**miRNA**	**Fold Change by Microarray**	**Fold Change by qRT-PCR**
EOC 13.31	miR-137	-9.9	-38.3
	miR-222	137	4153
EOC 20	miR-137	-1.7	-19.6
	miR-222	113	290
C8-B4	miR-137	-3.5	-10.7
	miR-222	17.9	102
NB41A3	miR-137	1.01	-3.7
	miR-222	ND	13.9
SK-N-FI	miR-137	11.1	11.4
	miR-222	ND	1.3
IMR-32	miR-137	-2.0	1.04
	miR-222	1.7	12.1

The fold change in expression was determined for six brain derived cell lines in comparison with their expression in the cell line N2a.

ND – miRNA was not detected using this methodology
