# Supplementary material for: Target labelling for the detection and profiling of microRNAs expressed in CNS tissue using microarrays
Source: BMC Biotechnol. 2006 Dec 12;6:47. doi: 10.1186/1472-6750-6-47 (PMC1713234; doi:10.1186/1472-6750-6-47)
Supplement: Additional File 1 — Oligonucleotide sequences of miRNA microarray probes. [file 1472-6750-6-47-S1.doc]

**Additional file**

**Table 1 -Oligonucleotide sequences of the miRNA microarray probes**

| **miRNA** | **Probe oligonucleotide sequence** |
| --- | --- |
| **osa-miR-396c** | TTTTTTTTTTTTTTTTTCCACAGCTTTCTTGAACTT |
| **cel-miR-84** | TTTTTTTTTTTTTTTTGAGGTAGTATGTAATATTGTA |
| **mmu-miR-131** | TTTTTTTTTTTTTTTTAAAGCTAGATAACCGAAAGT |
| **cel-miR-69** | TTTTTTTTTTTTTTTTCGAAAATTAAAAAGTGTAGA |
| **dme-miR-100** | TTTTTTTTTTTTTTTAACCCGTAAATCCGAACTTGTG |
| **miR-Lk-10** | TTTTTTTTTTTTTTTCGGCCTTAGTCGTCGGGGTGATT |
| **mmu-miR-344** | TTTTTTTTTTTTTTTTGATCTAGCCAAAGCCTGACTGT |
| **miR-Lk-23** | TTTTTTTTTTTTTTTGTGCTAGGATTGGGGCT |
| **mmu-let-7a-1** | TTTTTTTTTTTTTTTTGAGGTAGTAGGTTGTATAGTT |
| **mmu-miR-29b-1** | TTTTTTTTTTTTTTTTAGCACCATTTGAAATCAGTGT |
| **mmu-miR-345** | TTTTTTTTTTTTTTTTGCTGACCCCTAGTCCAGTGC |
| **mmu-miR-29a** | TTTTTTTTTTTTTTTCTAGCACCATCTGAAATCGGTT |
| **mmu-miR-206** | TTTTTTTTTTTTTTTTGGAATGTAAGGAAGTGTGTGG |
| **dme-miR-219** | TTTTTTTTTTTTTTTTGATTGTCCAAACGCAATTCTTG |
| **cel-lin-4** | TTTTTTTTTTTTTTTTCCCTGAGACCTCAAGTGTGA |
| **dme-miR-5** | TTTTTTTTTTTTTTTAAAGGAACGATCGTTGTGATATG |
| **osa-miR-396a** | TTTTTTTTTTTTTTTTTCCACAGCTTTCTTGAACTG |
| **cel-miR-65** | TTTTTTTTTTTTTTTTATGACACTGAAGCGTAACCGAA |
| **mmu-miR-190** | TTTTTTTTTTTTTTTTGATATGTTTGATATATTAGGT |
| **miR-302c** | TTTTTTTTTTTTTTTTAAGTGCTTCCATGTTTCAGTGG |
| **cel-miR-271** | TTTTTTTTTTTTTTTTCGCCGGGTGGGAAAGCATT |
| **miR-Lk-22c** | TTTTTTTTTTTTTTTTGATTCTGTGGGTGGTGGTGC |
| **mmu-miR-330** | TTTTTTTTTTTTTTTGCAAAGCACAGGGCCTGCAGAGA |
| **ath-miR-319c** | TTTTTTTTTTTTTTTTTGGACTGAAGGGAGCTCCT |
| **mmu-miR-1-1** | TTTTTTTTTTTTTTTTGGAATGTAAAGAAGTATGTA |
| **mmu-miR-27a** | TTTTTTTTTTTTTTTTTCACAGTGGCTAAGTTCCGC |
| **cel-miR-260** | TTTTTTTTTTTTTTTGTGATGTCGAACTCTTGTAG |
| **dme-miR-184** | TTTTTTTTTTTTTTTTGGACGGAGAACTGATAAGGGC |
| **cel-miR-75** | TTTTTTTTTTTTTTTTTAAAGCTACCAACCGGCTTCA |
| **mmu-miR-128b** | TTTTTTTTTTTTTTTTCACAGTGAACCGGTCTCTTTC |
| **mmu-miR-290** | TTTTTTTTTTTTTTTCTCAAACTATGGGGGCACTTTTT |
| **cel-miR-68** | TTTTTTTTTTTTTTTTCGAAGACTCAAAAGTGTAGA |
| **osa-miR-168b** | TTTTTTTTTTTTTTTAGGCTTGGTGCAGCTCGGGAA |
| **cel-miR-64** | TTTTTTTTTTTTTTTTATGACACTGAAGCGTTACCGAA |
| **cel-miR-265** | TTTTTTTTTTTTTTTTGAGGGAGGAAGGGTGGTAT |
| **mmu-miR-30b** | TTTTTTTTTTTTTTTTGTAAACATCCTACACTCAGC |
| **mmu-miR-207** | TTTTTTTTTTTTTTTGCTTCTCCTGGCTCTCCTCCCTC |
| **dme-miR-iab-4-5p** | TTTTTTTTTTTTTTTACGTATACTGAATGTATCCTGA |
| **miR-332** | TTTTTTTTTTTTTTTGGTCCCACCAGAGTCGCCA |
| **cel-miR-37** | TTTTTTTTTTTTTTTTCACCGGGTGAACACTTGCAGT |
| **dme-miR-276a*** | TTTTTTTTTTTTTTTCAGCGAGGTATAGAGTTCCTACG |
| **cel-miR-239a** | TTTTTTTTTTTTTTTTTTGTACTACACATAGGTACTGG |
| **mmu-miR-138-1** | TTTTTTTTTTTTTTTAGCTGGTGTTGTGAATC |
| **mmu-miR-141** | TTTTTTTTTTTTTTTAACACTGTCTGGTAAAGATGG |
| **mmu-miR-144** | TTTTTTTTTTTTTTTTACAGTATAGATGATGTACTAG |
| **miR-Lk-16** | TTTTTTTTTTTTTTTATAACGTTGAAAGATGGCATC |
| **mmu-miR-30e** | TTTTTTTTTTTTTTTTGTAAACATCCTTGACTGGA |
| **osa-miR-160d** | TTTTTTTTTTTTTTTTGCCTGGCTCCCTGTATGCCA |
| **mmu-miR-106a** | TTTTTTTTTTTTTTTCAAAGTGCTAACAGTGCAGGTA |
| **mirR-348** | TTTTTTTTTTTTTTTTGCCCACCCTTTACCCCACTCCAGT |
| **ebv-miR-BART2** | TTTTTTTTTTTTTTTTATTTTCTGCATTCGCCCTTGC |
| **cel-miR-229** | TTTTTTTTTTTTTTTAATGACACTGGTTATCTTTTCCATCGT |
| **cel-miR-42** | TTTTTTTTTTTTTTTCACCGGGTTAACATCTACAG |
| **mmu-miR-184** | TTTTTTTTTTTTTTTTGGACGGAGAACTGATAAGGGT |
| **ath-miR-158a** | TTTTTTTTTTTTTTTTCCCAAATGTAGACAAAGCA |
| **dme-miR-278** | TTTTTTTTTTTTTTTTCGGTGGGACTTTCGTCCGTTT |
| **cel-miR-244** | TTTTTTTTTTTTTTTTCTTTGGTTGTACAAAGTGGTATG |
| **mmu-miR-204** | TTTTTTTTTTTTTTTTTCCCTTTGTCATCCTATGCCTG |
| **osa-miR-395b** | TTTTTTTTTTTTTTTGTGAAGTGTTTGGGGGAACTC |
| **dme-miR-315** | TTTTTTTTTTTTTTTTTTTGATTGTTGCTCAGAAAGC |
| **osa-miR-160f** | TTTTTTTTTTTTTTTTGCCTGGCTCCCTGAATGCCA |
| **cel-miR-45** | TTTTTTTTTTTTTTTTGACTAGAGACACATTCAGCT |
| **rno-miR-352** | TTTTTTTTTTTTTTTAGAGTAGTAGGTTGCATAGTA |
| **miR-Lk-20** | TTTTTTTTTTTTTTTACTGGACTTGGAGTCAAAAGG |
| **mmu-miR-7b** | TTTTTTTTTTTTTTTTGGAAGACTTGTGATTTTGTT |
| **miR-Lk-30** | TTTTTTTTTTTTTTTGACCTGAGAGGGTGATCGGCCAC |
| **mmu-miR-142-5p** | TTTTTTTTTTTTTTTCATAAAGTAGAAAGCACTAC |
| **cel-miR-67** | TTTTTTTTTTTTTTTTCACAACCTCCTAGAAAGAGTAGA |
| **mmu-miR-199a** | TTTTTTTTTTTTTTTCCCAGTGTTCAGACTACCTGTTC |
| **mmu-miR-194-1** | TTTTTTTTTTTTTTTTGTAACAGCAACTCCATGTGGA |
| **osa-miR-399g** | TTTTTTTTTTTTTTTTGCCAAAGGAGATTTGCCCGG |
| **cel-miR-237** | TTTTTTTTTTTTTTTTCCCTGAGAATTCTCGAACAGCTT |
| **cel-miR-72** | TTTTTTTTTTTTTTTAGGCAAGATGTTGGCATAGC |
| **osa-miR-167d** | TTTTTTTTTTTTTTTTGAAGCTGCCAGCATGATCTG |
| **osa-miR-171a** | TTTTTTTTTTTTTTTTGATTGAGCCGCGCCAATATC |
| **cel-mir-46** | TTTTTTTTTTTTTTTTGTCATGGAGTCGCTCTCTTCA |
| **cel-miR-55** | TTTTTTTTTTTTTTTTACCCGTATAAGTTTCTGCTGAG |
| **cel-miR-259** | TTTTTTTTTTTTTTTAAATCTCATCCTAATCTGGTA |
| **ath-miR-157d** | TTTTTTTTTTTTTTTTGACAGAAGATAGAGAGCAC |
| **osa-miR-169q** | TTTTTTTTTTTTTTTTAGCCAAGGAGACTGCCCATG |
| **mmu-miR-92-1** | TTTTTTTTTTTTTTTTATTGCACTTGTCCCGGCCTG |
| **cel-miR-2** | TTTTTTTTTTTTTTTTATCACAGCCAGCTTTGATGTGC |
| **mmu-miR-200b** | TTTTTTTTTTTTTTTTAATACTGCCTGGTAATGATGAC |
| **cel-miR-228** | TTTTTTTTTTTTTTTAATGGCACTGCATGAATTCACGG |
| **rno-miR-129*** | TTTTTTTTTTTTTTTAAGCCCTTACCCCAAAAAGCAT |
| **mmu-miR-218-1** | TTTTTTTTTTTTTTTTTGTGCTTGATCTAACCATGT |
| **mmu-miR-321** | TTTTTTTTTTTTTTTTAAGCCAGGGATTGTGGGTTC |
| **miR-Lk-5** | TTTTTTTTTTTTTTTGTGCTAGGGATTGGGGCTTG |
| **mmu-miR-292-5p** | TTTTTTTTTTTTTTTACTCAAACTGGGGGCTCTTTTG |
| **gga-miR-1b** | TTTTTTTTTTTTTTTTGGAATGTTAAGAAGTATGTA |
| **mmu-miR-325** | TTTTTTTTTTTTTTTCCTAGTAGGTGCTCAGTAAGTGT |
| **hsa-miR-369** | TTTTTTTTTTTTTTTAATAATACATGGTTGATCTTT |
| **ath-miR-319a** | TTTTTTTTTTTTTTTTTGGACTGAAGGGAGCTCCC |
| **cel-miR-66** | TTTTTTTTTTTTTTTCATGACACTGATTAGGGATGTGA |
| **mmu-miR-217** | TTTTTTTTTTTTTTTTACTGCATCAGGAACTGACTGGAT |
| **dme-miR-276b*** | TTTTTTTTTTTTTTTCAGCGAGGTATAGAGTTCCTACG |
| **cel-miR-261** | TTTTTTTTTTTTTTTTAGCTTTTTAGTTTTCACG |
| **cel-miR-77** | TTTTTTTTTTTTTTTTTCATCAGGCCATAGCTGTCCA |
| **cel-miR-73** | TTTTTTTTTTTTTTTTGGCAAGATGTAGGCAGTTCAGT |
| **cel-miR-38** | TTTTTTTTTTTTTTTTCACCGGGAGAAAAACTGGAGT |
| **dme-miR-309** | TTTTTTTTTTTTTTTGCACTGGGTAAAGTTTGTCCTA |
| **cel-miR-241** | TTTTTTTTTTTTTTTTGAGGTAGGTGCGAGAAATGA |
| **miR-302d** | TTTTTTTTTTTTTTTTAAGTGCTTCCATGTTTGAGTGT |
| **mmu-miR-183** | TTTTTTTTTTTTTTTTATGGCACTGGTAGAATTCACTG |
| **mmu-miR-181a** | TTTTTTTTTTTTTTTAACATTCAACGCTGTCGGTGAGT |
| **miR-Lk-11** | TTTTTTTTTTTTTTTAGGAGCACAGCTGGGTATCTAAGT |
| **cel-miR-269** | TTTTTTTTTTTTTTTGGCAAGACTCTGGCAAAACT |
| **cel-miR-359** | TTTTTTTTTTTTTTTTCACTGGTCTTTCTCTGACGA |
| **gga-miR-205b** | TTTTTTTTTTTTTTTCCTTCATTCCACCGGAATCTG |
| **osa-miR159a** | TTTTTTTTTTTTTTTTTTGGATTGAAGGGAGCTCTG |
| **dme-miR-276b** | TTTTTTTTTTTTTTTTAGGAACTTAATACCGTGCTCT |
| **cel-miR-61** | TTTTTTTTTTTTTTTTGACTAGAACCGTTACTCATCTC |
| **rno-miR-151*** | TTTTTTTTTTTTTTTTCGAGGAGCTCACAGTCTAGTA |
| **dme-miR-314** | TTTTTTTTTTTTTTTTATTCGAGCCAATAAGTTCGG |
| **miR-196b** | TTTTTTTTTTTTTTTTGCCCACCCTTTACCCCACTCCAGT |
| **mmu-miR-100** | TTTTTTTTTTTTTTTAACCCGTAGATCCGAACTTGTG |
| **mmu-miR-302** | TTTTTTTTTTTTTTTTAAGTGCTTCCATGTTTTGGTGA |
| **mmu-miR-215** | TTTTTTTTTTTTTTTATGACCTATGATTTGACAGAC |
| **dme-miR-87** | TTTTTTTTTTTTTTTTTGAGCAAAATTTCAGGTGTG |
| **mmu-miR-187** | TTTTTTTTTTTTTTTTCGTGTCTTGTGTTGCAGCCGG |
| **mmu-miR-153-1** | TTTTTTTTTTTTTTTTTGCATAGTCACAAAAGTGA |
| **miR-Lk-17** | TTTTTTTTTTTTTTTCGAGCCGTCGTAGACCACGACGTT |
| **mmu-miR-24-1** | TTTTTTTTTTTTTTTTGGCTCAGTTCAGCAGGAACAG |
| **miR-Lk-1** | TTTTTTTTTTTTTTTGGACTGCCTCAGCTGTGC |
| **dme-miR-31a** | TTTTTTTTTTTTTTTTGGCAAGATGTCGGCATAGCTGA |
| **miR-Lk-13** | TTTTTTTTTTTTTTTCGTAGACCCGAAACCGGGTGAC |
| **ebv-miR-BHRF1-3** | TTTTTTTTTTTTTTTTAACGGGAAGTGTGTAAGCACAC |
| **gga-miR-124b-1** | TTTTTTTTTTTTTTTTTAAGGCACGCAGTGAATGCCA |
| **dme-miR-317** | TTTTTTTTTTTTTTTTGAACACAGCTGGTGGTATCCAGT |
| **mmu-miR-297-1** | TTTTTTTTTTTTTTTATGTATGTGTGCATGTGCATG |
| **miR-Lk-24** | TTTTTTTTTTTTTTTAGCCAATGGTGCGAAGCTA |
| **has-miR-370** | TTTTTTTTTTTTTTTGCCTGCTGGGGTGGAACCTGG |
| **miR-196c** | TTTTTTTTTTTTTTTGGGCTGGGCCGGTCGGGC |
| **mmu-miR-134** | TTTTTTTTTTTTTTTTGTGACTGGTTGACCAGAGGG |
| **mmu-miR-140** | TTTTTTTTTTTTTTTAGTGGTTTTACCCTATGGTAG |
| **mmu-miR-125b-1** | TTTTTTTTTTTTTTTTCCCTGAGACCCTAACTTGTGA |
| **dme-miR-9c** | TTTTTTTTTTTTTTTTCTTTGGTATTCTAGCTGTAGA |
| **cel-miR-71** | TTTTTTTTTTTTTTTTGAAAGACATGGGTAGTGA |
| **mmu-miR-301** | TTTTTTTTTTTTTTTCAGTGCAATAGTATTGTCAAAGC |
| **mmu-miR-17-5p** | TTTTTTTTTTTTTTTCAAAGTGCTTACAGTGCAGGTAGT |
| **dme-miR-282** | TTTTTTTTTTTTTTTAATCTAGCCTCTACTAGGCTTTGTCTGT |
| **cel-miR-74** | TTTTTTTTTTTTTTTTGGCAAGAAATGGCAGTCTACA |
| **miR-154*** | TTTTTTTTTTTTTTTAATCATACACGGTTGACCTATT |
| **miR-334** | TTTTTTTTTTTTTTTTAAACGGTGCAGAGATGTG |
| **mmu-miR-152** | TTTTTTTTTTTTTTTTCAGTGCATGACAGAACTTGG |
| **mmu-miR-137** | TTTTTTTTTTTTTTTTATTGCTTAAGAATACGCGTAG |
| **dme-miR-263a** | TTTTTTTTTTTTTTTGTTAATGGCACTGGAAGAATTCAC |
| **mmu-miR-135a-1** | TTTTTTTTTTTTTTTTATGGCTTTTTATTCCTATGTGA |
| **dme-miR-133** | TTTTTTTTTTTTTTTTTGGTCCCCTTCAACCAGCTGT |
| **cel-miR-240** | TTTTTTTTTTTTTTTTACTGGCCCCCAAATCTTCGCT |
| **ath-miR-158b** | TTTTTTTTTTTTTTTCCCCAAATGTAGACAAAGCA |
| **mmu-miR-154** | TTTTTTTTTTTTTTTTTAGGTTATCCGTGTTGCCTTCG |
| **osa-miR-169d** | TTTTTTTTTTTTTTTTAGCCAAGGATGAATTGCCGG |
| **hsa-miR-374** | TTTTTTTTTTTTTTTTTATAATACAACCTGATAAGTG |
| **osa-miR-398a** | TTTTTTTTTTTTTTTTGTGTTCTCAGGTCACCCCTT |
| **cel-miR-49** | TTTTTTTTTTTTTTTAAGCACCACGAGAAGCTGCAGA |
| **mmu-miR-101** | TTTTTTTTTTTTTTTTACAGTACTGTGATAACTGA |
| **cel-miR-60** | TTTTTTTTTTTTTTTTATTATGCACATTTTCTAGTTCA |
| **osa-miR-166k** | TTTTTTTTTTTTTTTTCGGACCAGGCTTCAATCCCT |
| **mmu-miR-210** | TTTTTTTTTTTTTTTCTGTGCGTGTGACAGCGGCTG |
| **mmu-miR-98** | TTTTTTTTTTTTTTTTGAGGTAGTAAGTTGTATTGTT |
| **dme-miR-308** | TTTTTTTTTTTTTTTAATCACAGGATTATACTGTGAG |
| **dme-miR-6-1** | TTTTTTTTTTTTTTTTATCACAGTGGCTGTTCTTTTT |
| **rno-miR-347** | TTTTTTTTTTTTTTTTTGTCCCTCTGGGTCGCCA |
| **mmu-miR-200c** | TTTTTTTTTTTTTTTAATACTGCCGGGTAATGATGGA |
| **cel-miR-236** | TTTTTTTTTTTTTTTTAATACTGTCAGGTAATGACGCT |
| **osa-miR-162a** | TTTTTTTTTTTTTTTTCGATAAACCTCTGCATCCAG |
| **osa-miR-399i** | TTTTTTTTTTTTTTTTGCCAAAGGAGAGCTGCCCTG |
| **dme-miR-287** | TTTTTTTTTTTTTTTTGTGTTGAAAATCGTTTGCAC |
| **mmu-miR-188** | TTTTTTTTTTTTTTTCATCCCTTGCATGGTGGAGGGT |
| **mmu-miR-326** | TTTTTTTTTTTTTTTCCTCTGGGCCCTTCCTCCAGT |
| **osa-miR-395c** | TTTTTTTTTTTTTTTGTGAAGTGCTTGGGGGAACTC |
| **mmu-miR-185** | TTTTTTTTTTTTTTTTGGAGAGAAAGGCAGTTC |
| **osa-miR-399d** | TTTTTTTTTTTTTTTTGCCAAAGGAGAGTTGCCCTG |
| **mmu-miR-15a** | TTTTTTTTTTTTTTTTAGCAGCACATAATGGTTTGTG |
| **osa-miR-164e** | TTTTTTTTTTTTTTTTGGAGAAGCAGGGCACGTGAG |
| **mmu-miR-126** | TTTTTTTTTTTTTTTTCGTACCGTGAGTAATAATGC |
| **mmu-miR-331** | TTTTTTTTTTTTTTTGCCCCTGGGCCTATCCTAGAA |
| **dme-miR-279** | TTTTTTTTTTTTTTTTGACTAGATCCACACTCATTAA |
| **mmu-miR-292-3p** | TTTTTTTTTTTTTTTAAGTGCCGCCAGGTTTTGAGTGT |
| **miR-Lk-38** | TTTTTTTTTTTTTTTCAGACAGGGTGTACATGACCTT |
| **dme-miR-305** | TTTTTTTTTTTTTTTATTGTACTTCATCAGGTGCTCTG |
| **miR-Lk-34** | TTTTTTTTTTTTTTTAGACCCACCAGGCGTTCGGCC |
| **osa-miR-172b** | TTTTTTTTTTTTTTTGGAATCTTGATGATGCTGCAT |
| **mmu-miR-106b** | TTTTTTTTTTTTTTTTAAAGTGCTGACAGTGCAGAT |
| **mmu-miR-129-1** | TTTTTTTTTTTTTTTCTTTTTGCGGTCTGGGCTTGCT |
| **mmu-miR-29c** | TTTTTTTTTTTTTTTTAGCACCATTTGAAATCGGTTA |
| **mmu-miR-10a-1** | TTTTTTTTTTTTTTTTACCCTGTAGATCCGAATTTGTG |
| **mmu-miR-127** | TTTTTTTTTTTTTTTTCGGATCCGTCTGAGCTTGGCT |
| **dme-miR-iab-4-3p** | TTTTTTTTTTTTTTTCGGTATACCTTCAGTATACGTAAC |
| **dme-miR-7** | TTTTTTTTTTTTTTTTGGAAGACTAGTGATTTTGTTGT |
| **hsa-miR-182*** | TTTTTTTTTTTTTTTTGGTTCTAGACTTGCCAACTA |
| **mmu-miR-149** | TTTTTTTTTTTTTTTTCTGGCTCCGTGTCTTCACTCC |
| **mmu-miR-181b-1** | TTTTTTTTTTTTTTTAACATTCATTGCTGTCGGTGGGTT |
| **hsa-miR-371** | TTTTTTTTTTTTTTTGTGCCGCCATCTTTTGAGTGT |
| **dme-miR-31b** | TTTTTTTTTTTTTTTTGGCAAGATGTCGGAATAGCTG |
| **ath-miR-163** | TTTTTTTTTTTTTTTTTGAAGAGGACTTGGAACTTCGAT |
| **osa-miR-172a** | TTTTTTTTTTTTTTTAGAATCTTGATGATGCTGCAT |
| **cel-miR-392** | TTTTTTTTTTTTTTTTATCATCGATCACGTGTGATGA |
| **osa-miR-399a** | TTTTTTTTTTTTTTTTGCCAAAGGAGAATTGCCCTG |
| **mmu-miR-26b** | TTTTTTTTTTTTTTTTTCAAGTAATTCAGGATAGGTT |
| **mmu-miR-324-5p** | TTTTTTTTTTTTTTTCGCATCCCCTAGGGCATTGGTGT |
| **mmu-miR-139** | TTTTTTTTTTTTTTTTCTACAGTGCACGTGTCT |
| **mmu-miR-335** | TTTTTTTTTTTTTTTTCAAGAGCAATAACGAAAAATGT |
| **cel-miR-43** | TTTTTTTTTTTTTTTTATCACAGTTTACTTGCTGTCGC |
| **osa-miR-156j** | TTTTTTTTTTTTTTTTGACAGAAGAGAGTGAGCAC |
| **dme-miR-210** | TTTTTTTTTTTTTTTTTGTGCGTGTGACAGCGGCTA |
| **miR-Lk-27** | TTTTTTTTTTTTTTTCCGTAGGCCGTTGAAGCGATC |
| **mmu-let-7i** | TTTTTTTTTTTTTTTTGAGGTAGTAGTTTGTGCT |
| **cel-miR-87** | TTTTTTTTTTTTTTTGTGAGCAAAGTTTCAGGTGT |
| **dme-miR-92a** | TTTTTTTTTTTTTTTCATTGCACTTGTCCCGGCCTAT |
| **mmu-miR-216** | TTTTTTTTTTTTTTTTAATCTCAGCTGGCAACTGTG |
| **mmu-miR-295** | TTTTTTTTTTTTTTTAAAGTGCTACTACTTTTGAGTCT |
| **mmu-let-7e** | TTTTTTTTTTTTTTTTGAGGTAGGAGGTTGTATAGT |
| **miR-Lk-28** | TTTTTTTTTTTTTTTTTAGTATGGTTGCCTTCCAA |
| **dme-let-7** | TTTTTTTTTTTTTTTTGAGGTAGTAGGTTGTATAGT |
| **ath-miR-161** | TTTTTTTTTTTTTTTTTGAAAGTGACTACATCGGGG |
| **dme-miR-303** | TTTTTTTTTTTTTTTTTTAGGTTTCACAGGAAACTGGT |
| **dme-miR-2a-1** | TTTTTTTTTTTTTTTTATCACAGCCAGCTTTGATGAGC |
| **ath-miR-157a** | TTTTTTTTTTTTTTTTTGACAGAAGATAGAGAGCAC |
| **miR-Lk-3** | TTTTTTTTTTTTTTTGGTTGATAGGTCGGGGGTGTAA |
| **dme-miR-310** | TTTTTTTTTTTTTTTTATTGCACACTTCCCGGCCTTT |
| **dme-miR-10** | TTTTTTTTTTTTTTTACCCTGTAGATCCGAATTTGT |
| **mmu-miR-299** | TTTTTTTTTTTTTTTTGGTTTACCGTCCCACATACAT |
| **cel-miR-58** | TTTTTTTTTTTTTTTTGAGATCGTTCAGTACGGCAAT |
| **cel-miR-56** | TTTTTTTTTTTTTTTTACCCGTAATGTTTCCGCTGAG |
| **cel-miR-234** | TTTTTTTTTTTTTTTTTATTGCTCGAGAATACCCTT |
| **dme-miR-184*** | TTTTTTTTTTTTTTTCCTTATCATTCTCTCGCCCCG |
| **miR-Lk-25** | TTTTTTTTTTTTTTTTGTTGAAAAAGCATGGGATG |
| **mmu-miR-32** | TTTTTTTTTTTTTTTTATTGCACATTACTAAGTTGC |
| **mmu-miR-351** | TTTTTTTTTTTTTTTTCCCTGAGGAGCCCTTTGAGCCTG |
| **mmu-miR-199a*** | TTTTTTTTTTTTTTTTACAGTAGTCTGCACATTGGTT |
| **mmu-miR-130a** | TTTTTTTTTTTTTTTCAGTGCAATGTTAAAAGGGC |
| **dme-miR-33** | TTTTTTTTTTTTTTTAGGTGCATTGTAGTCGCATTG |
| **mmu-miR-205** | TTTTTTTTTTTTTTTTCCTTCATTCCACCGGAGTCTG |
| **cel-miR-273** | TTTTTTTTTTTTTTTTGCCCGTACTGTGTCGGCTG |
| **dme-miR-288** | TTTTTTTTTTTTTTTTTTCATGTCGATTTCATTTCATG |
| **cel-miR-251** | TTTTTTTTTTTTTTTTTAAGTAGTGGTGCCGCTCTTATT |
| **miR-Lk-36** | TTTTTTTTTTTTTTTCAAAGCAGCAGTATCGCCT |
| **rno-miR-336** | TTTTTTTTTTTTTTTTCACCCTTCCATATCTAGTCT |
| **dme-miR-285** | TTTTTTTTTTTTTTTTAGCACCATTCGAAATCAGTGC |
| **osa-miR-399j** | TTTTTTTTTTTTTTTTGCCAAAGGAGAGTTGCCCTA |
| **osa-miR-397a** | TTTTTTTTTTTTTTTTCATTGAGTGCAGCGTTGATG |
| **hsa-miR-106a** | TTTTTTTTTTTTTTTAAAAGTGCTTACAGTGCAGGTAGC |
| **mmu-miR-341** | TTTTTTTTTTTTTTTTCGATCGGTCGGTCGGTCAGT |
| **mmu-miR-132** | TTTTTTTTTTTTTTTTAACAGTCTACAGCCATGGTCG |
| **hsa-miR-368** | TTTTTTTTTTTTTTTACATAGAGGAAATTCCACGTTT |
| **ath-miR-172e** | TTTTTTTTTTTTTTTGGAATCTTGATGATGCTGCAT |
| **miR-302a*** | TTTTTTTTTTTTTTTTAAACGTGGATGTACTTGCTTT |
| **mmu-let-7g** | TTTTTTTTTTTTTTTTGAGGTAGTAGTTTGTACAGT |
| **mmu-miR-291-5p** | TTTTTTTTTTTTTTTCATCAAAGTGGAGGCCCTCTCT |
| **cel-miR-356** | TTTTTTTTTTTTTTTTTGAGCAACGCGAACAAATCA |
| **mmu-miR-155** | TTTTTTTTTTTTTTTTTAATGCTAATTGTGATAGGGG |
| **dme-miR-34** | TTTTTTTTTTTTTTTTGGCAGTGTGGTTAGCTGGTTG |
| **mmu-miR-93** | TTTTTTTTTTTTTTTCAAAGTGCTGTTCGTGCAGGTAG |
| **miR-Lk-12** | TTTTTTTTTTTTTTTCAACCTTGGGATACCACCCTGTA |
| **mmu-miR-107** | TTTTTTTTTTTTTTTAGCAGCATTGTACAGGGCTATCA |
| **cel-miR-36** | TTTTTTTTTTTTTTTTCACCGGGTGAAAATTCGCATG |
| **dme-miR-11** | TTTTTTTTTTTTTTTCATCACAGTCTGAGTTCTTGC |
| **osa-miR159c** | TTTTTTTTTTTTTTTATTGGATTGAAGGGAGCTCCA |
| **cel-miR-247** | TTTTTTTTTTTTTTTTGACTAGAGCCTATTCTCTTCTT |
| **cel-miR-76** | TTTTTTTTTTTTTTTTTCGTTGTTGATGAAGCCTTGA |
| **ath-miR165a** | TTTTTTTTTTTTTTTTCGGACCAGGCTTCATCCCCC |
| **cel-miR-267** | TTTTTTTTTTTTTTTCCCGTGAAGTGTCTGCTGCA |
| **mmu-miR-28** | TTTTTTTTTTTTTTTAAGGAGCTCACAGTCTATTGAG |
| **mmu-miR-181c** | TTTTTTTTTTTTTTTAACATTCAACCTGTCGGTGAGT |
| **cel-miR-272** | TTTTTTTTTTTTTTTTGTAGGCATGGGTGTTTG |
| **cel-miR-245** | TTTTTTTTTTTTTTTATTGGTCCCCTCCAAGTAGCTC |
| **mmu-miR-30c-1** | TTTTTTTTTTTTTTTTGTAAACATCCTACACTCTCAGC |
| **miR-302c*** | TTTTTTTTTTTTTTTTTTAACATGGGGGTACCTGCTG |
| **cel-miR-239b** | TTTTTTTTTTTTTTTTTGTACTACACAAAAGTACTG |
| **mmu-miR-23b** | TTTTTTTTTTTTTTTATCACATTGCCAGGGATTACCAC |
| **ebv-miR-BHRF1-1** | TTTTTTTTTTTTTTTTAACCTGATCAGCCCCGGAGTT |
| **miR-Lk-29** | TTTTTTTTTTTTTTTTCGTCCCGAGACCGATTATTT |
| **mmu-miR-219-1** | TTTTTTTTTTTTTTTTGATTGTCCAAACGCAATTCT |
| **cel-miR-355** | TTTTTTTTTTTTTTTTTTGTTTTAGCCTGAGCTATG |
| **mmu-miR-9-1** | TTTTTTTTTTTTTTTTCTTTGGTTATCTAGCTGTATGA |
| **dme-miR-275** | TTTTTTTTTTTTTTTTCAGGTACCTGAAGTAGCGCGCG |
| **cel-miR-264** | TTTTTTTTTTTTTTTGGCGGGTGGTTGTTGTTATG |
| **mmu-miR-342** | TTTTTTTTTTTTTTTTCTCACACAGAAATCGCACCCGTC |
| **mmu-miR-145** | TTTTTTTTTTTTTTTGTCCAGTTTTCCCAGGAATCCCTT |
| **cel-miR-79** | TTTTTTTTTTTTTTTATAAAGCTAGGTTACCAAAGCT |
| **gga-miR-222b** | TTTTTTTTTTTTTTTAACTACATCTGGCTACTGGGTCTC |
| **miR-Lk-26** | TTTTTTTTTTTTTTTTAAAGTGCTGACAGCTCAGATA |
| **mmu-miR-15b** | TTTTTTTTTTTTTTTTAGCAGCACATCATGGTTTACA |
| **mmu-miR-214** | TTTTTTTTTTTTTTTACAGCAGGCACAGACAGGCAG |
| **mmu-miR-208** | TTTTTTTTTTTTTTTATAAGACGAGCAAAAAGCTTGT |
| **dme-miR-13b-1** | TTTTTTTTTTTTTTTTATCACAGCCATTTTGACGAGT |
| **mmu-miR-193** | TTTTTTTTTTTTTTTTAACTGGCCTACAAAGTCCCAG |
| **mmu-miR-20** | TTTTTTTTTTTTTTTTAAAGTGCTTATAGTGCAGGTAG |
| **cel-miR-354** | TTTTTTTTTTTTTTTACCTTGTTTGTTGCTGCTCCT |
| **mmu-miR-142-3p** | TTTTTTTTTTTTTTTTGTAGTGTTTCCTACTTTATGG |
| **mmu-let-7d*** | TTTTTTTTTTTTTTTCTATACGACCTGCTGCCTTTCT |
| **cel-miR-124** | TTTTTTTTTTTTTTTTAAGGCACGCGGTGAATGCCA |
| **osa-miR-169h** | TTTTTTTTTTTTTTTTAGCCAAGGATGACTTGCCTG |
| **dme-miR-125** | TTTTTTTTTTTTTTTTCCCTGAGACCCTAACTTGTGA |
| **cel-miR-232** | TTTTTTTTTTTTTTTTAAATGCATCTTAACTGCGGTGA |
| **mmu-miR-30a** | TTTTTTTTTTTTTTTCTTTCAGTCGGATGTTTGCAGC |
| **mmu-miR-291-3p** | TTTTTTTTTTTTTTTAAAGTGCTTCCACTTTGTGTGCC |
| **cel-miR-1** | TTTTTTTTTTTTTTTTGGAATGTAAAGAAGTATGTA |
| **dme-miR-12** | TTTTTTTTTTTTTTTTGAGTATTACATCAGGTACTGGT |
| **cel-miR-82** | TTTTTTTTTTTTTTTTGAGATCATCGTGAAAGCCAGT |
| **mmu-miR-146** | TTTTTTTTTTTTTTTTGAGAACTGAATTCCATGGGTT |
| **mmu-miR-133a-1** | TTTTTTTTTTTTTTTTTGGTCCCCTTCAACCAGCTGT |
| **cel-miR-249** | TTTTTTTTTTTTTTTTCACAGGACTTTTGAGCGTTGC |
| **miR-302b** | TTTTTTTTTTTTTTTTAAGTGCTTCCATGTTTTAGTAG |
| **mmu-miR-17-3p** | TTTTTTTTTTTTTTTACTGCAGTGAGGGCACTTGT |
| **miR-Lk-19** | TTTTTTTTTTTTTTTCAAAGTGCTTACAGTTCAGGTAG |
| **osa-miR-159e** | TTTTTTTTTTTTTTTATTGGATTGAAGGGAGCTCCT |
| **cel-miR-47** | TTTTTTTTTTTTTTTTGTCATGGAGGCGCTCTCTTCA |
| **hsa-miR-196b** | TTTTTTTTTTTTTTTTAGGTAGTTTCCTGTTGTTGG |
| **cel-miR-56*** | TTTTTTTTTTTTTTTTGGCGGATCCATTTTGGGTTGTA |
| **miR-Lk-35** | TTTTTTTTTTTTTTTTTAGATGAGATAACAGGTTTCT |
| **cel-miR-231** | TTTTTTTTTTTTTTTTAAGCTCGTGATCAACAGGCAGAA |
| **cel-miR-53** | TTTTTTTTTTTTTTTCACCCGTACATTTGTTTCCGTGCT |
| **cel-miR-80** | TTTTTTTTTTTTTTTTGAGATCATTAGTTGAAAGCCGA |
| **cel-miR-258** | TTTTTTTTTTTTTTTGGTTTTGAGAGGAATCCTTTT |
| **cel-miR-250** | TTTTTTTTTTTTTTTTCACAGTCAACTGTTGGCATGG |
| **gga-miR-18b** | TTTTTTTTTTTTTTTTAAGGTGCATCTAGTGCAGTTA |
| **miR-Lk-14** | TTTTTTTTTTTTTTTTACCGCATAATGTTGAAAGATGG |
| **cel-miR-235** | TTTTTTTTTTTTTTTTATTGCACTCTCCCCGGCCTGA |
| **ebv-miR-BART1** | TTTTTTTTTTTTTTTTCTTAGTGGAAGTGACGTGCT |
| **osa-miR-394** | TTTTTTTTTTTTTTTTTGGCATTCTGTCCACCTCC |
| **cel-miR-70** | TTTTTTTTTTTTTTTTAATACGTCGTTGGTGTTTCCAT |
| **dme-miR-311** | TTTTTTTTTTTTTTTTATTGCACATTCACCGGCCTGA |
| **cel-miR-230** | TTTTTTTTTTTTTTTGTATTAGTTGTGCGACCAGGAGA |
| **mmu-miR-202** | TTTTTTTTTTTTTTTAGAGGTATAGCGCATGGGAAGA |
| **hsa-miR-372** | TTTTTTTTTTTTTTTAAAGTGCTGCGACATTTGAGCGT |
| **cel-mir-360** | TTTTTTTTTTTTTTTTGACCGTAATCCCGTTCACAA |
| **cel-lsy-6** | TTTTTTTTTTTTTTTTTTTGTATGAGACGCATTTCG |
| **dme-miR-312** | TTTTTTTTTTTTTTTTATTGCACTTGAGACGGCCTGA |
| **mmu-miR-221** | TTTTTTTTTTTTTTTAGCTACATTGTCTGCTGGGTTT |
| **osa-miR-167a** | TTTTTTTTTTTTTTTTGAAGCTGCCAGCATGATCTA |
| **osa-miR-168a** | TTTTTTTTTTTTTTTTCGCTTGGTGCAGATCGGGAC |
| **miR-Lk-21** | TTTTTTTTTTTTTTTCCTGGGTGCCAAGTTTTGAC |
| **hsa-miR-198** | TTTTTTTTTTTTTTTGGTCCAGAGGGGAGATAGG |
| **dme-miR-284** | TTTTTTTTTTTTTTTGAAGTCAGCAACTTGATTCCAGCAATTG |
| **mmu-miR-143** | TTTTTTTTTTTTTTTTGAGATGAAGCACTGTAGCTCA |
| **rno-miR-20*** | TTTTTTTTTTTTTTTACTGCATTACGAGCACTTACA |
| **ath-miR-172d** | TTTTTTTTTTTTTTTAGAATCTTGATGATGCTGCAG |
| **dme-miR-9a** | TTTTTTTTTTTTTTTTCTTTGGTTATCTAGCTGTATGA |
| **cel-miR-81** | TTTTTTTTTTTTTTTTGAGATCATCGTGAAAGCTAGT |
| **cel-miR-41** | TTTTTTTTTTTTTTTTCACCGGGTGAAAAATCACCTA |
| **mmu-miR-199b** | TTTTTTTTTTTTTTTCCCAGTGTTTAGACTACCTGTTC |
| **cel-miR-57** | TTTTTTTTTTTTTTTTACCCTGTAGATCGAGCTGTGTGT |
| **osa-miR-166a** | TTTTTTTTTTTTTTTTCGGACCAGGCTTCATTCCCC |
| **mmu-miR-222** | TTTTTTTTTTTTTTTAGCTACATCTGGCTACTGGGTCT |
| **dme-miR-1** | TTTTTTTTTTTTTTTTGGAATGTAAAGAAGTATGGAG |
| **mmu-miR-103-1** | TTTTTTTTTTTTTTTAGCAGCATTGTACAGGGCTATGA |
| **mmu-miR-192** | TTTTTTTTTTTTTTTCTGACCTATGAATTGACA |
| **mmu-miR-293** | TTTTTTTTTTTTTTTAGTGCCGCAGAGTTTGTAGTGT |
| **mmu-miR-33** | TTTTTTTTTTTTTTTGTGCATTGTAGTTGCATTG |
| **dme-miR-281** | TTTTTTTTTTTTTTTTGTCATGGAATTGCTCTCTTTGT |
| **cel-miR-86** | TTTTTTTTTTTTTTTTAAGTGAATGCTTTGCCACAGTC |
| **cel-miR-238** | TTTTTTTTTTTTTTTTTTGTACTCCGATGCCATTCAGA |
| **osa-miR-156l** | TTTTTTTTTTTTTTTCGACAGAAGAGAGTGAGCATA |
| **dme-miR-306*** | TTTTTTTTTTTTTTTGGGGGTCACTCTGTGCCTGTGC |
| **osa-miR-399k** | TTTTTTTTTTTTTTTTGCCAAAGGAAATTTGCCCCG |
| **mmu-miR-298** | TTTTTTTTTTTTTTTGGCAGAGGAGGGCTGTTCTTCC |
| **miR-Lk-31** | TTTTTTTTTTTTTTTGTACTTAGTAGAGCAGCCAC |
| **mmu-miR-18** | TTTTTTTTTTTTTTTTAAGGTGCATCTAGTGCAGATA |
| **miR-Lk-22b** | TTTTTTTTTTTTTTTTGATTCCGTGGGTGGTGGTGC |
| **hsa-miR-154*** | TTTTTTTTTTTTTTTAATCATACACGGTTGACCTATT |
| **osa-miR-399h** | TTTTTTTTTTTTTTTTGCCAAAGGAGACTTGCCCAG |
| **dme-miR-306** | TTTTTTTTTTTTTTTTCAGGTACTTAGTGACTCTCAA |
| **mmu-miR-191** | TTTTTTTTTTTTTTTCAACGGAATCCCAAAAGCAGCT |
| **hsa-miR-95** | TTTTTTTTTTTTTTTTTCAACGGGTATTTATTGAGCA |
| **mmu-miR-324-3p** | TTTTTTTTTTTTTTTCCACTGCCCCAGGTGCTGCTGG |
| **mmu-miR-224** | TTTTTTTTTTTTTTTTAAGTCACTAGTGGTTCCGTTTA |
| **dme-miR-9b** | TTTTTTTTTTTTTTTTCTTTGGTGATTTTAGCTGTATG |
| **cel-miR-40** | TTTTTTTTTTTTTTTTCACCGGGTGTACATCAGCTAA |
| **mmu-miR-34a** | TTTTTTTTTTTTTTTTGGCAGTGTCTTAGCTGGTTGTT |
| **mmu-miR-328** | TTTTTTTTTTTTTTTCTGGCCCTCTCTGCCCTTCCGT |
| **cel-miR-50** | TTTTTTTTTTTTTTTTGATATGTCTGGTATTCTTGGGTT |
| **mmu-miR-350** | TTTTTTTTTTTTTTTTTCACAAAGCCCATACACTTTCAC |
| **dme-miR-307** | TTTTTTTTTTTTTTTTCACAACCTCCTTGAGTGAG |
| **dme-miR-318** | TTTTTTTTTTTTTTTTCACTGGGCTTTGTTTATCTCA |
| **dme-miR-263b** | TTTTTTTTTTTTTTTCTTGGCACTGGGAGAATTCAC |
| **ebv-miR-BHRF1-2** | TTTTTTTTTTTTTTTTATCTTTTGCGGCAGAAATTGAA |
| **dme-miR-79** | TTTTTTTTTTTTTTTTAAAGCTAGATTACCAAAGCAT |
| **mmu-let-7f-1** | TTTTTTTTTTTTTTTTGAGGTAGTAGATTGTATAGTT |
| **miR-196c** | TTTTTTTTTTTTTTTTCCACCTAGCCGGGAGAACCA |
| **cel-miR-35** | TTTTTTTTTTTTTTTTCACCGGGTGGAAACTAGCAGT |
| **cel-miR-243** | TTTTTTTTTTTTTTTCGGTACGATCGCGGCGGGATATC |
| **mmu-miR-294** | TTTTTTTTTTTTTTTAAAGTGCTTCCCTTTTGTGTGT |
| **dme-miR-14** | TTTTTTTTTTTTTTTTCAGTCTTTTTCTCTCTCCTA |
| **mmu-miR-21** | TTTTTTTTTTTTTTTTAGCTTATCAGACTGATGTTGA |
| **cel-miR-39** | TTTTTTTTTTTTTTTTCACCGGGTGTAAATCAGCTTG |
| **mmu-miR-148a** | TTTTTTTTTTTTTTTTCAGTGCACTACAGAACTTTGT |
| **osa-miR-395p** | TTTTTTTTTTTTTTTGTGAAGCGTTTGGGGGAAATC |
| **dme-mir-8** | TTTTTTTTTTTTTTTTAATACTGTCAGGTAAAGATGTC |
| **mmu-miR-26a-1** | TTTTTTTTTTTTTTTTTCAAGTAATCCAGGATAGGCT |
| **mmu-miR-201** | TTTTTTTTTTTTTTTTACTCAGTAAGGCATTGTTCT |
| **osa-miR-159f** | TTTTTTTTTTTTTTTCTTGGATTGAAGGGAGCTCTA |
| **dme-mir-283** | TTTTTTTTTTTTTTTTAAATATCAGCTGGTAATTCT |
| **mmu-miR-212** | TTTTTTTTTTTTTTTTAACAGTCTCCAGTCACGGCC |
| **mmu-miR-9-1*** | TTTTTTTTTTTTTTTTAAAGCTAGATAACCGAAAGT |
| **mmu-miR-19b-1** | TTTTTTTTTTTTTTTTGTGCAAATCCATGCAAAACTGA |
| **mmu-miR-31** | TTTTTTTTTTTTTTTAGGCAAGATGCTGGCATAGCTG |
| **osa-miR-164c** | TTTTTTTTTTTTTTTTGGAGAAGCAGGGTACGTGCA |
| **dme-miR-2c** | TTTTTTTTTTTTTTTTATCACAGCCAGCTTTGATGGGC |
| **miR-Lk-32** | TTTTTTTTTTTTTTTGGCGTAAAGGGAGCGTACGCGGAT |
| **miR-Lk-15** | TTTTTTTTTTTTTTTCGGTTCATACCCGAAGGGTCGCAA |
| **cel-miR-233** | TTTTTTTTTTTTTTTTTGAGCAATGCGCATGTGCGGGA |
| **miR-Lk-7** | TTTTTTTTTTTTTTTACTACGAATGATAACATCCGTGG |
| **miR-Lk-33** | TTTTTTTTTTTTTTTGGCGTAAAGGGAGCGTACGCGGAT |
| **osa-miR-164d** | TTTTTTTTTTTTTTTTGGAGAAGCAGGGCACGTGCT |
| **dme-miR-281-1*** | TTTTTTTTTTTTTTTAAGAGAGCTGTCCGTCGACAGT |
| **cel-miR-358** | TTTTTTTTTTTTTTTCAATTGGTATCCCTGTCAAGG |
| **osa-miR-319a** | TTTTTTTTTTTTTTTTTGGACTGAAGGGTGCTCCC |
| **mmu-miR-320** | TTTTTTTTTTTTTTTAAAAGCTGGGTTGAGAGGGCGAA |
| **mmu-miR-196a-1** | TTTTTTTTTTTTTTTTAGGTAGTTTCATGTTGTTGG |
| **mmu-miR-34c** | TTTTTTTTTTTTTTTAGGCAGTGTAGTTAGCTGATTGC |
| **osa-miR-160e** | TTTTTTTTTTTTTTTTGCCTGGCTCCCTGTATGCCG |
| **cel-miR-256** | TTTTTTTTTTTTTTTTGGAATGCATAGAAGACTGTA |
| **mmu-miR-182** | TTTTTTTTTTTTTTTTTTGGCAATGGTAGAACTCACA |
| **miR-Lk-22a** | TTTTTTTTTTTTTTTTGATTCGGTGGGTGGTGGTGC |
| **mmu-miR-200a** | TTTTTTTTTTTTTTTTAACACTGTCTGGTAACGATGT |
| **osa-miR171b** | TTTTTTTTTTTTTTTTGATTGAGCCGTGCCAATATC |
| **mmu-miR-Lk-351** | TTTTTTTTTTTTTTTTCCCTGAGAGCCCTTTGAGCCTGT |
| **osa-miR171g** | TTTTTTTTTTTTTTTGAGGTGAGCCGAGCCAATATC |
| **mmu-miR-340** | TTTTTTTTTTTTTTTTCCGTCTCAGTTACTTTATAGCC |
| **mmu-miR-130b** | TTTTTTTTTTTTTTTCAGTGCAATGATGAAAGGGCAT |
| **cel-miR-51** | TTTTTTTTTTTTTTTTACCCGTAGCTCCTATCCATGTT |
| **mmu-let-7b** | TTTTTTTTTTTTTTTTGAGGTAGTAGGTTGTGTGGTT |
| **cel-miR-78** | TTTTTTTTTTTTTTTTGGAGGCCTGGTTGTTTGTGC |
| **mmu-miR-133b** | TTTTTTTTTTTTTTTTTGGTCCCCTTCAACCAGCTA |
| **cel-miR-357** | TTTTTTTTTTTTTTTTAAATGCCAGTCGTTGCAGGA |
| **miR-Lk-4** | TTTTTTTTTTTTTTTATTTCAGGTGAAGTTTCAAGAGTC |
| **rno-miR-343** | TTTTTTTTTTTTTTTTCTCCCTCCGTGTGCCCAGTT |
| **cel-miR-90** | TTTTTTTTTTTTTTTTGATATGTTGTTTGAATGCCCC |
| **miR-302b*** | TTTTTTTTTTTTTTTACTTTAACATGGAAGTGCTTTCT |
| **hsa-miR-373*** | TTTTTTTTTTTTTTTACTCAAAATGGGGGCGCTTTCC |
| **cel-miR-257** | TTTTTTTTTTTTTTTGAGTATCAGGAGTACCCAGTGA |
| **osa-miR-169p** | TTTTTTTTTTTTTTTTAGCCAAGGACAAACTTGCCGG |
| **cel-miR-254** | TTTTTTTTTTTTTTTTGCAAATCTTTCGCGACTGTAGG |
| **gga-let-7k** | TTTTTTTTTTTTTTTTGAGGTAGTAGATTGAATAGTT |
| **dme-miR-286** | TTTTTTTTTTTTTTTTGACTAGACCGAACACTCGTGCT |
| **osa-miR-399e** | TTTTTTTTTTTTTTTTGCCAAAGGAGATTTGCCCAG |
| **mmu-miR-150** | TTTTTTTTTTTTTTTTCTCCCAACCCTTGTACCAGTG |
| **hsa-miR-373** | TTTTTTTTTTTTTTTGAAGTGCTTCGATTTTGGGGTGT |
| **cel-miR-59** | TTTTTTTTTTTTTTTTCGAATCGTTTATCAGGATGATG |
| **dme-miR-280** | TTTTTTTTTTTTTTTTGTATTTACGTTGCATATGAAATGATA |
| **dme-miR-289** | TTTTTTTTTTTTTTTTAAATATTTAAGTGGAGCCTGCGACT |
| **ebv-miR-BHRF1-2*** | TTTTTTTTTTTTTTTAAATTCTGTTGCAGCAGATAGC |
| **rno-miR-7-1*** | TTTTTTTTTTTTTTTCAACAAATCACAGTCTGCCATA |
| **hsa-miR-105-1** | TTTTTTTTTTTTTTTTCAAATGCTCAGACTCCTGT |
| **mmu-miR-329** | TTTTTTTTTTTTTTTAACACACCCAGCTAACCTTTTT |
| **mmu-miR-338** | TTTTTTTTTTTTTTTTCCAGCATCAGTGATTTTGTTGA |
| **cel-miR-270** | TTTTTTTTTTTTTTTGGCATGATGTAGCAGTGGAG |
| **dme-miR-277** | TTTTTTTTTTTTTTTTAAATGCACTATCTGGTACGACA |
| **cel-miR-44** | TTTTTTTTTTTTTTTTGACTAGAGACACATTCAGCT |
| **miR-Lk-6a** | TTTTTTTTTTTTTTTCAAAGTGCTGTTAGTGCAGGTAG |
| **dme-miR-276a** | TTTTTTTTTTTTTTTTAGGAACTTCATACCGTGCTCT |
| **cel-miR-83** | TTTTTTTTTTTTTTTTAGCACCATATAAATTCAGTAA |
| **mmu-miR-16-1** | TTTTTTTTTTTTTTTTAGCAGCACGTAAATATTGGCG |
| **osa-miR-159d** | TTTTTTTTTTTTTTTATTGGATTGAAGGGAGCTCCG |
| **rno-miR-349** | TTTTTTTTTTTTTTTCAGCCCTGCTGTCTTAACCTCT |
| **mmu-miR-211** | TTTTTTTTTTTTTTTTTCCCTTTGTCATCCTTTGCCT |
| **mmu-miR-125a** | TTTTTTTTTTTTTTTTCCCTGAGACCCTTTAACCTGTG |
| **osa-miR-398b** | TTTTTTTTTTTTTTTTGTGTTCTCAGGTCGCCCCTG |
| **cel-miR-246** | TTTTTTTTTTTTTTTTTACATGTTTCGGGTAGGAGCT |
| **mmu-miR-7-1** | TTTTTTTTTTTTTTTTGGAAGACTAGTGATTTTGTT |
| **mmu-miR-22** | TTTTTTTTTTTTTTTAAGCTGCCAGTTGAAGAACTGT |
| **rno-miR-140*** | TTTTTTTTTTTTTTTTACCACAGGGTAGAACCACGGACA |
| **dme-bantam** | TTTTTTTTTTTTTTTTGAGATCATTTTGAAAGCTGATT |
| **osa-miR-172c** | TTTTTTTTTTTTTTTTGAATCTTGATGATGCTGCAC |
| **osa-miR-395a** | TTTTTTTTTTTTTTTGTGAAGTATTTGGGGGAACTC |
| **mmu-miR-23a** | TTTTTTTTTTTTTTTATCACATTGCCAGGGATTTCC |
| **cel-miR-252** | TTTTTTTTTTTTTTTTAAGTAGTAGTGCCGCAGGTAAC |
| **mmu-miR-10b** | TTTTTTTTTTTTTTTCCCTGTAGAACCGAATTTGTGT |
| **hsa-miR-220** | TTTTTTTTTTTTTTTCCACACCGTATCTGACACTTT |
| **miR-196a** | TTTTTTTTTTTTTTTTTAAACGGTGCAGAGATGTG |
| **mmu-miR-25** | TTTTTTTTTTTTTTTCATTGCACTTGTCTCGGTCTGA |
| **mmu-miR-101b** | TTTTTTTTTTTTTTTTACAGTACTGTGATAGCTGAAG |
| **hsa-miR-367** | TTTTTTTTTTTTTTTAATTGCACTTTAGCAATGGTGA |
| **mmu-miR-30a*** | TTTTTTTTTTTTTTTTGTAAACATCCTCGACTGGAAGC |
| **cel-miR-48** | TTTTTTTTTTTTTTTTGAGGTAGGCTCAGTAGATGCGA |
| **mmu-miR-124a-1** | TTTTTTTTTTTTTTTTTAAGGCACGCGGTGAATGCCA |
| **cel-miR-63** | TTTTTTTTTTTTTTTTATGACACTGAAGCGAGTTGGAAA |
| **mmu-miR-296** | TTTTTTTTTTTTTTTAGGGCCCCCCCTCAATCCTGT |
| **dme-miR-4** | TTTTTTTTTTTTTTTATAAAGCTAGACAACCATTGA |
| **osa-miR-169f** | TTTTTTTTTTTTTTTTAGCCAAGGATGACTTGCCTA |
| **cel-miR-54** | TTTTTTTTTTTTTTTTACCCGTAATCTTCATAATCCGAG |
| **cel-let-7** | TTTTTTTTTTTTTTTTGAGGTAGTAGGTTGTATAGTT |
| **osa-miR-166j** | TTTTTTTTTTTTTTTTCGGATCAGGCTTCATTCCTC |
| **mmu-miR-189** | TTTTTTTTTTTTTTTGTGCCTACTGAGCTGATATCAGT |
| **mmu-miR-195** | TTTTTTTTTTTTTTTTAGCAGCACAGAAATATTGGC |
| **mmu-miR-135b** | TTTTTTTTTTTTTTTTATGGCTTTTCATTCCTATGTG |
| **osa-miR162b** | TTTTTTTTTTTTTTTTCGATAAGCCTCTGCATCCAG |
| **mmu-miR-322** | TTTTTTTTTTTTTTTAAACATGAAGCGCTGCAACA |
| **osa-miR-169a** | TTTTTTTTTTTTTTTCAGCCAAGGATGACTTGCCGA |
| **cel-miR-242** | TTTTTTTTTTTTTTTTTGCGTAGGCCTTTGCTTCGA |
| **miR-Lk-8** | TTTTTTTTTTTTTTTATGCAAGTCGAGCTTGAAGTTTC |
| **dme-miR-316** | TTTTTTTTTTTTTTTTGTCTTTTTCCGCTTACTGGCG |
| **cel-miR-268** | TTTTTTTTTTTTTTTGGCAAGAATTAGAAGCAGTTTGGT |
| **mmu-miR-186** | TTTTTTTTTTTTTTTCAAAGAATTCTCCTTTTGGGCTT |
| **cel-miR-253** | TTTTTTTTTTTTTTTCACACCTCACTAACACTGACC |
| **mmu-miR-148b** | TTTTTTTTTTTTTTTTCAGTGCATCACAGAACTTTGT |
| **mmu-miR-136** | TTTTTTTTTTTTTTTACTCCATTTGTTTTGATGATGGA |
| **hsa-miR-147** | TTTTTTTTTTTTTTTGTGTGTGGAAATGCTTCTGC |
| **rno-miR-333** | TTTTTTTTTTTTTTTGTGGTGTGCTAGTTACTTTT |
| **osa-miR-169b** | TTTTTTTTTTTTTTTCAGCCAAGGATGACTTGCCGG |
| **dme-miR-92b** | TTTTTTTTTTTTTTTAATTGCACTAGTCCCGGCCTGC |
| **mmu-miR-122a** | TTTTTTTTTTTTTTTTGGAGTGTGACAATGGTGTTTGT |
| **mmu-miR-213** | TTTTTTTTTTTTTTTACCATCGACCGTTGATTGTACC |
| **cel-miR-62** | TTTTTTTTTTTTTTTTGATATGTAATCTAGCTTACAG |
| **cel-miR-52** | TTTTTTTTTTTTTTTCACCCGTACATATGTTTCCGTGCT |
| **hsa-miR-101-1** | TTTTTTTTTTTTTTTTACAGTACTGTGATAACTGAAG |
| **ath-miR-170** | TTTTTTTTTTTTTTTTGATTGAGCCGTGTCAATATC |
| **mmu-miR-27b** | TTTTTTTTTTTTTTTTTCACAGTGGCTAAGTTCTG |
| **ath-miR-173** | TTTTTTTTTTTTTTTTTCGCTTGCAGAGAGAAATCAC |
| **miR-Lk-37** | TTTTTTTTTTTTTTTATGCCTGTCGGTTACTGCCTGCT |
| **mmu-miR-96** | TTTTTTTTTTTTTTTTTTGGCACTAGCACATTTTTGCT |
| **mmu-miR-323** | TTTTTTTTTTTTTTTGCACATTACACGGTCGACCTCT |
| **mmu-miR-128a** | TTTTTTTTTTTTTTTTCACAGTGAACCGGTCTCTTTT |
| **osa-miR-393** | TTTTTTTTTTTTTTTTCCAAAGGGATCGCATTGATC |
| **dme-miR-13a** | TTTTTTTTTTTTTTTTATCACAGCCATTTTGATGAGT |
| **mmu-miR-19a** | TTTTTTTTTTTTTTTTGTGCAAATCTATGCAAAACTGA |
| **mmu-miR-300** | TTTTTTTTTTTTTTTTATGCAAGGGCAAGCTCTCTTC |
| **mmu-miR-30d** | TTTTTTTTTTTTTTTTGTAAACATCCCCGACTGGAAG |
| **hsa-miR-197** | TTTTTTTTTTTTTTTTTCACCACCTTCTCCACCCAGC |
| **cel-miR-34** | TTTTTTTTTTTTTTTAGGCAGTGTGGTTAGCTGGTTG |
| **miR-Lk-9** | TTTTTTTTTTTTTTTATCGTTATTATCGATGGCGTGA |
| **osa-miR-166g** | TTTTTTTTTTTTTTTTCGGACCAGGCTTCATTCCTC |
| **osa-miR-164a** | TTTTTTTTTTTTTTTTGGAGAAGCAGGGCACGTGCA |
| **miR-like** | TTTTTTTTTTTTTTTGGTCCCACCAGAGTCGCCA |
| **dme-miR-2b-1** | TTTTTTTTTTTTTTTTATCACAGCCAGCTTTGAGGAGC |
| **cel-miR-266** | TTTTTTTTTTTTTTTAGGCAAGACTTTGGCAAAGC |
| **osa-miR-156k** | TTTTTTTTTTTTTTTTGACAGAAGAGAGAGAGCACA |
| **miR-Lk-6b** | TTTTTTTTTTTTTTTCAAAGTGCTGTTATTGCAGG |
| **mmu-miR-151** | TTTTTTTTTTTTTTTCTAGACTGAGGCTCCTTGAGG |
| **mmu-miR-99a** | TTTTTTTTTTTTTTTACCCGTAGATCCGATCTTGT |
| **mmu-miR-99b** | TTTTTTTTTTTTTTTCACCCGTAGAACCGACCTTGCG |
| **mmu-let-7d** | TTTTTTTTTTTTTTTAGAGGTAGTAGGTTGCATAGT |
| **dme-miR-3** | TTTTTTTTTTTTTTTTCACTGGGCAAAGTGTGTCTCA |
| **mmu-miR-346** | TTTTTTTTTTTTTTTTGTCTGCCCGAGTGCCTGCCTCT |
| **mmu-miR-203** | TTTTTTTTTTTTTTTTGAAATGTTTAGGACCACTAG |
| **dme-miR-304** | TTTTTTTTTTTTTTTTAATCTCAATTTGTAAATGTGAG |
| **cel-miR-262** | TTTTTTTTTTTTTTTGTTTCTCGATGTTTTCTGAT |
| **mmu-let-7c-1** | TTTTTTTTTTTTTTTTGAGGTAGTAGGTTGTATGGTT |
| **mmu-miR-126*** | TTTTTTTTTTTTTTTCATTATTACTTTTGGTACGCG |
| **rno-miR-327** | TTTTTTTTTTTTTTTCCTTGAGGGGCATGAGGGT |
| **cel-miR-248** | TTTTTTTTTTTTTTTTACACGTGCACGGATAACGCTCA |
| **osa-miR397b** | TTTTTTTTTTTTTTTTTATTGAGTGCAGCGTTGATG |
| **mmu-miR-339** | TTTTTTTTTTTTTTTTCCCTGTCCTCCAGGAGCTCA |
| **miR-Lk-2** | TTTTTTTTTTTTTTTGTGTCCTAAGGTGAGCTCAG |
| **cel-miR-353** | TTTTTTTTTTTTTTTCAATTGCCATGTGTTGGTATT |
| **dme-miR-313** | TTTTTTTTTTTTTTTTATTGCACTTTTCACAGCCCGA |
| **cel-miR-227** | TTTTTTTTTTTTTTTAGCTTTCGACATGATTCTGAAC |
| **cel-miR-85** | TTTTTTTTTTTTTTTTACAAAGTATTTGAAAAGTCGTGC |
| **miR-Lk-18** | TTTTTTTTTTTTTTTGCCGTCGTCGACGAGTGCACTT |
| **mmu-miR-34b** | TTTTTTTTTTTTTTTTAGGCAGTGTAATTAGCTGATTG |
| **dme-miR-124** | TTTTTTTTTTTTTTTTAAGGCACGCGGTGAATGCCAAG |
| **mmu-miR-337** | TTTTTTTTTTTTTTTTTCAGCTCCTATATGATGCCTTT |
| **dme-miR-274** | TTTTTTTTTTTTTTTTTTTGTGACCGACACTAACGGGTAAT |
| **mmu-miR-223** | TTTTTTTTTTTTTTTTGTCAGTTTGTCAAATACCCC |
