# Supplementary material for: Target labelling for the detection and profiling of microRNAs expressed in CNS tissue using microarrays
Source: BMC Biotechnol. 2006 Dec 12;6:47. doi: 10.1186/1472-6750-6-47 (PMC1713234; doi:10.1186/1472-6750-6-47)
Supplement: Additional File 2 — Schematic comparison between the direct labelling strategy (labelled-cDNA) and labelling upon linear amplification (labelled-aRNA). [file 1472-6750-6-47-S2.doc]

**miRNA**

**Poly A tailed miRNA**

**1. Poly (A) tailing of miRNA**

**2. Reverse transcription of tailed miRNA**

**dT**

**Capture sequence**

*

*

*

*

*

*

*

*

*

**3. Tagged cDNA hybridization**

**4. Dendrimer hybridization**

**Probe oligo**

**microarray**

**Complement to capture sequence**

**3. Second strand synthesis**

**4. In vitro transcription**

**incorporating modified**

**nucleotides**

**5. Coupling of dye molecules**

*

*

*

*

*

*

*

*

**6. Microarray hybridization**

**DNA dendrimer**

***Labelled-aRNA synthesis***

***Labelled-cDNA synthesis***
